# Hybrid fabrication of multimodal intracranial implants for electrophysiology and local drug delivery†

**DOI:** 10.1039/d1mh01855h

**Published:** 2022-04-21

**Authors:** Johannes Gurke, Tobias E. Naegele, Sam Hilton, Roberto Pezone, Vincenzo F. Curto, Damiano G. Barone, Emil J. W. List-Kratochvil, Alejandro Carnicer-Lombarte, George G. Malliaras

**Affiliations:** University of Cambridge, Electrical Engineering Division 9 JJ Thomson Ave Cambridge CB3 0FA UK gm603@cam.ac.uk; University of Cambridge, School of Clinical Medicine, Department of Clinical Neurosciences, Cambridge Biomedical Campus Cambridge CB2 0QQ UK; Humboldt-Universität zu Berlin, Department of Chemistry and of Physics and IRIS Adlershof, Hybrid Devices Group, Zum Großen Windkanal 2 12489 Berlin Germany; Helmholtz-Zentrum für Materialien und Energie GmbH, Hahn-Meitner-Platz 1 14109 Berlin Germany

## Abstract

New fabrication approaches for mechanically flexible implants hold the key to advancing the applications of neuroengineering in fundamental neuroscience and clinic. By combining the high precision of thin film microfabrication with the versatility of additive manufacturing, we demonstrate a straight-forward approach for the prototyping of intracranial implants with electrode arrays and microfluidic channels. We show that the implant can modulate neuronal activity in the hippocampus through localized drug delivery, while simultaneously recording brain activity by its electrodes. Moreover, good implant stability and minimal tissue response are seen one-week post-implantation. Our work shows the potential of hybrid fabrication combining different manufacturing techniques in neurotechnology and paves the way for a new approach to the development of multimodal implants.

## Abstract 2

New fabrication approaches for mechanically flexible implants hold the key to advancing the applications of neuroengineering in fundamental neuroscience and clinic.

## Graphical abstract

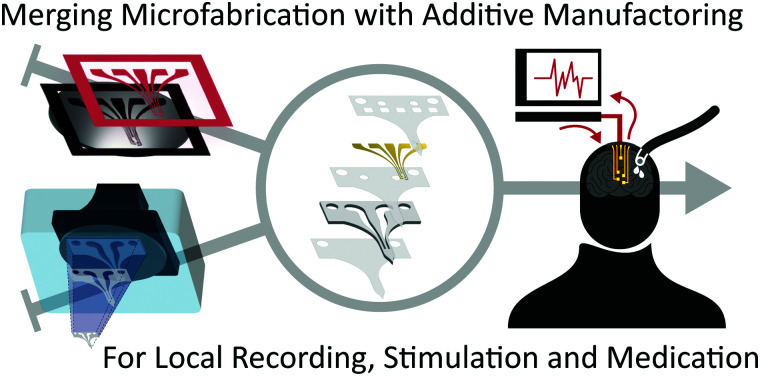

**New concepts**

In this manuscript we demonstrate a new fabrication approach for neural implants that combines additive manufacturing with thin film microfabrication. We use this approach to build and validate an implant capable of electrical stimulation and recording of the brain and of localised drug delivery. Multimodal implants are of interest for fundamental neuroscience studies and bioelectronic medicine and we show that a hybrid fabrication process approach yields implants with good performance and stability. Furthermore, this work shows that additive manufacturing methods have advanced enough to be considered for neurotechnology applications, either in combination with other techniques, or on their own.

## Introduction

Mechanically flexible, multimodal implants have emerged in the past decade as powerful tools for studying and controlling the brain.^1–3^ Mechanical flexibility can lead to decreased damage to the surrounding tissue and minimise the foreign body reaction (FBR), leading to significant improvements in implant lifetime and performance.^4–6^ At the same time, the combination of electrical, biochemical, optical and other modalities can offer a better understanding of the brain and lead to better ways to control its states.^7,8^ Different materials and manufacturing techniques have been used to produce such implants. One prominent examples is silicon micromachining, used to develop injectable implants equipped with a variety of sensing and actuation modalities.^9^ Glass fibre drawing was used to fabricate thin penetrating implants that integrate neural recording, optical stimulation and drug delivery capabilities.^10^ Finally, microfabrication on elastomers was used to demonstrate cortical implants with electrophysiology devices and microfluidic channels for drug delivery.^11^

Rapid prototyping *via* computer aided design and manufacturing (CAD + M) is attracting a great deal of attention for rapid translation of academic research to the clinic.^12–15^ The digital nature of these techniques enables the use of patient data, *e.g. via* MRI or CT scans, to customise implant design.^16^ Examples include stereolithography (SLA) and digital light processing (DLP).^17,18^ These techniques make use of vat polymerizable resins^19,20^ and allow the affordable processing of materials with different properties, including mechanical flexibility.^21,22^ In microfluidic systems, the use of CAD + M is replacing classic fabrication techniques including micro moulding and micro stamping.^23–29^ A few early examples of neuronal implants have been demonstrated, exploring the use of various 3D printing techniques.^15,25,30,31^

Rapid prototyping, however, faces some formidable limitations, such as poor spatial resolution and limited materials gamut. There is, therefore, scope for exploring the combination of traditional microfabrication techniques, that allow the fabrication of for instance high definition electrodes, with CAD + M that yield *e.g.* bespoke microfluidic channels. Here, we demonstrate such a hybrid fabrication process that facilitates a multimodal intracranial implant capable of performing electrophysiology measurements and drug delivery. The implant is mechanically flexible and interfaces with a 3D-printed headstage that provides electrical and fluidic connections. We validate the implant in an *in vivo* model, showing electrical stimulation, recording of hippocampal electrophysiological activity, and local administration of two exemplary drugs. Our work shows that the combination of different fabrication techniques can be successfully used to produce multimodal implants for interfacing with the brain.

## Results

### Implant design

**Fig. 1:**
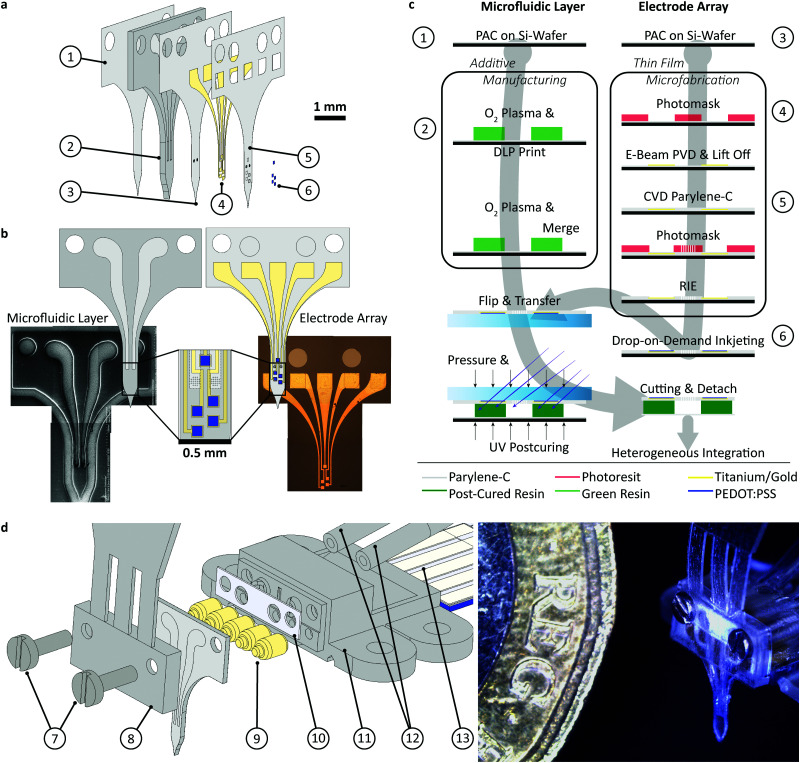
Hybrid fabrication of the multimodal intracranial implant: (a) exploded view of the implant (① to ⑥) and assembled headstage for acute *in vivo* experiments (⑦ to 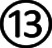): ① bottom PAC layer (2 μm), ② 3D printed, flexible body with microfluidic system (200 μm), ③ middle PAC layer (2 μm) with μ-fluidic in- and outlet, ④ 10 nm titanium-100 nm gold electrodes ⑤, top PAC layer (2 μm) with etched μ-fluidic in- and outlets as well as electrical contacts, ⑥ Drop-on-demand inkjet-printed PEDOT:PSS layer (77 ± 11 nm), ⑦ fastener M0.6 × 5 mm, ⑧ 3D printedsocket for acute *in vivo* experiments, ⑨ pogo pins, ⑩ medical-grade double-sided adhesive tape (85 μm), 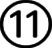 3D printed fluidic and electric interface with M0.6 thread, 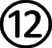 1/32′′ polyethylene tubing (0.8 mm OD, 0.4 mm ID) and 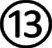 tailored FPC cable (5 ways, 1 mm pitch); (b) left: technical drawing and SEM picture of 3D printed body showing the μ-fluidic channels (top view) and right: technical drawing and optical micrograph of the Au layer (top view). (c) Convergent hybrid fabrication schema, combining additive manufacturing and thin-film microfabrication; (d) exploded view and microscopy picture of the assembly.

**Fig. 2:**
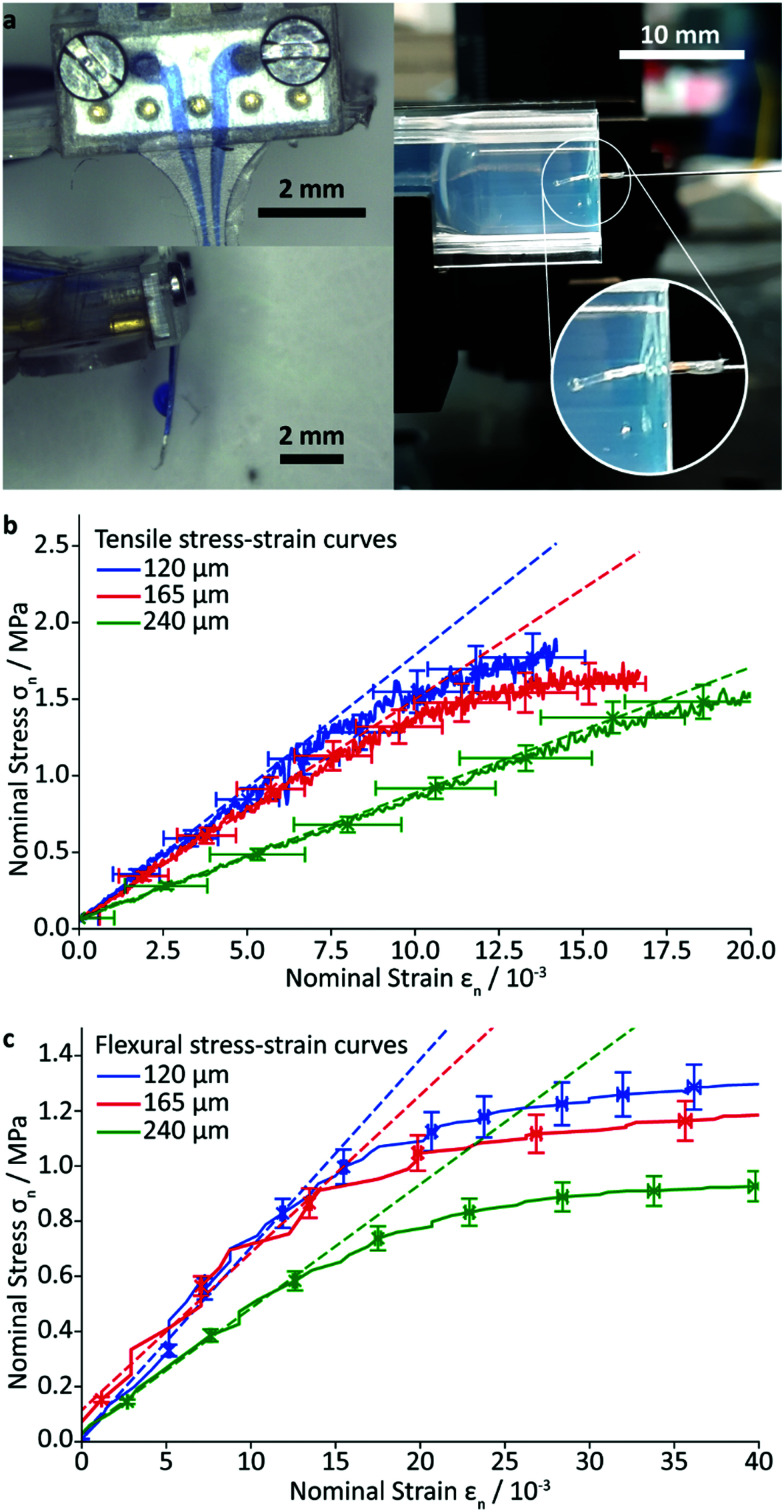
Characterisation of the implant: (a) left: test and visualisation of the drug delivery *via* the microfluidic system with methylene blue coloured water, and right: qualitative implant insertion test into 0.6% m/v agarose gel; (b) tensile and (c) flexural stress strain curves of rectangular specimens with variation of the 3D printed resin thickness in between two PAC layer (2 μm each). The dashed lines are fits to the linear regions of the curves at small strain. To improve readability, the error bars are only displayed for selected data points.

**Fig. 3:**
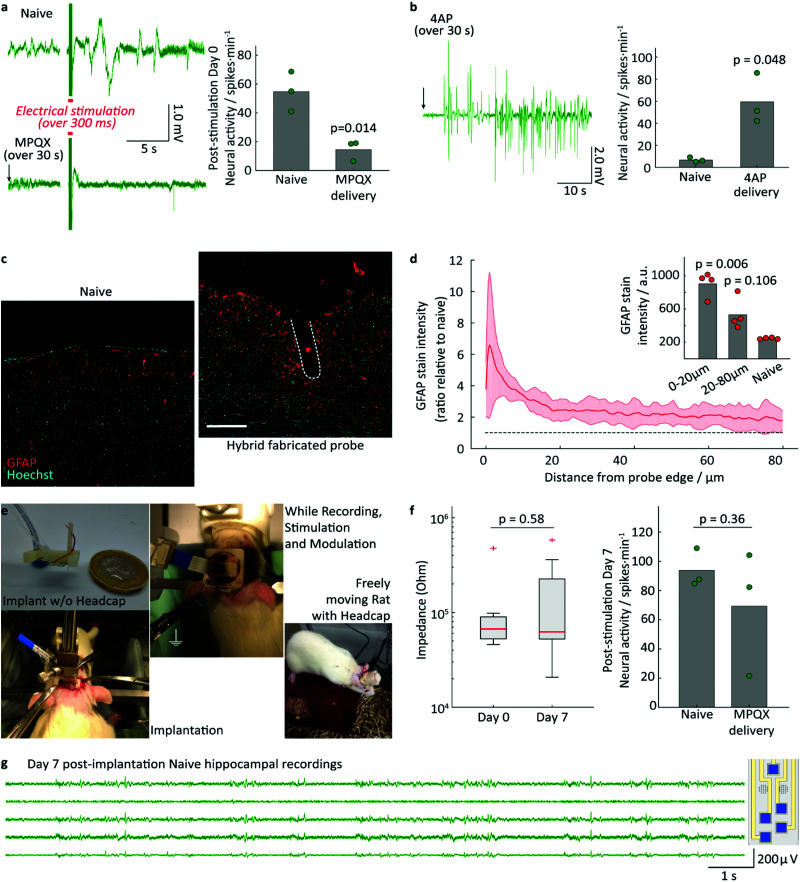
*In vivo* validation of hybrid fabricated implant functionality and biocompatibility: (a) left: representative neural recording from rat hippocampus, showing large amplitude interictal-like spiking activity in response to electrical stimulation (red line) in naive animals. These could be supressed through the delivery of MPQX prior to electrical stimulation (MPQX delivered locally *via* the implant, over 30 s, ending at black arrow); right: quantification of neural interictal-like spike activity per minute upon electric stimulation with and w/o MPQX delivery (*N* = 3 rats, first minute after electrical stimulation); (b) left: representative neural recording from rat hippocampus following the localized delivery of the convulsant 4AP through the implant (over 30 s, ending at black arrow); right: quantification of neural interictal spike activity show the increase in neural activity caused by 4AP (*N* = 3 rats, first two minutes after drug delivery). Statistical comparison in a and b done *via* paired *t*-test. Circles in plots represent average spikes per minute, bar represents average across all rats; (c) immunofluorescence images of contralateral hemisphere (Naive) as a control and of the tissue response to hybrid fabricated implants 7 days post-implantation. Tissue is fluorescently labelled for reactive astrocytes (GFAP, red) and cell nuclei (Hoechst, cyan). Approximate location of implant indicated by white dashed line. scale bar: 100 μm; (d) quantification of astrocytic response based on GFAP intensity profile, calculated as fold change with respect to contralateral control. Solid line corresponds to average GFAP intensity for *N* = 4 rats, shaded envelope corresponds to standard deviation. Black dashed line indicates GFAP intensity levels in contralateral naive tissue; inset: Plot of average GFAP intensity (arbitrary units) in tissue immediately surrounding the implant (0–10 μm) and more peripheral tissue (10–80 μm), compared to contralateral tissue (naive). Statistical comparison in c and d done *via* Bonferroni-corrected paired t-test to the non-implanted control group. Circles in plot represent average value for each tissue region group per rat, bar represents average across all rats; (e) setup for long-term implantation; (f) performance of the modules in long-term implantation experiments (left) electrodes and (right) drug delivery, (*N* = 3 rats); (g) day 7 post-implantation naive HP recordings.

The device assembly was design-engineered at a generic, digital 3D model of a rat skull and brain (see electronic ESI† Fig. S1). The assembly consists of a flexible implant (Fig. 1a ① to ⑥) and a headstage (Fig. 1d ⑦ to 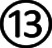). The printed main implant body (Fig. 1a ②) is made of a commercially available, photocurable polyacrylate^32^ resin layer (200 μm) sandwiched between two Parylene-C (PAC) layers (2 μm bottom ① and 4 μm top ③ and ⑤). Two μ-fluidic channels in the printed body ② allow localized drug delivery (see Fig. 1b, left). The top PAC layer ③/⑤ holds the thin-film gold electronics (④ and ⑥). Near the implant's tip, five gold electrodes (100 × 100 μm^2^, see Fig. 1b, right) with a 77 ± 11 nm thick PEDOT:PSS coating ⑥ are arranged for neural recording and electrical stimulation. Further, two sets of 22 holes (15 μm ID) are etched into the PAC ③/⑤, connecting the μ-fluidic channels with the targeted tissue. At base of the implant, two wells for M0.6 fasteners, an etched hole fitting to the male, μ-fluidic interface and five titanium/gold connector pads (500 × 500 μm^2^) for electrical interfacing. The overall length of the implant amounts to 5.8 mm. The interface (Fig. 1d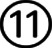) consists out of five wells, receiving pogo pins ⑨, two threads for the M0.6 screws and male, μ-fluidic connectors towards the implant as well as a female connector for a 1/32′′ polyethylene tube 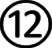 and a tailored flexible cable 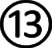. To ensure a tight-fitting, a ratchet was printed into the female fluidic connector. The μ-fluidic connection between headstage and implant is sealed by medical-grade, double-sided adhesive tape (85 μm thickness, ⑩). The socket ⑧ consists of a shim that aligns and fastens the implant onto the headstage, and a handle for the surgeon. Here, two variations of the socket has been developed. An unadorned version for acute *in vivo* experiments as shown in Fig. 1 and 2 as well as a more robust version for long term experiment. Later allows the mounting of a headcap as show in Fig. 3 and in detail in the ESI.† A line of fracture allows to remove the handle after implantation. The assembly is designed using a rat model, so that the recording/stimulation pads as well as site of delivery are located in the hippocampus.

### Implant fabrication

Details on the implant and headstage design as well as their heterogeneous integration are provided in the ESI† (Fig. S1 and S6). As shown in Fig. 1c, the implant fabrication starts with chemical vapor deposition (CVD) of 2 μm of PAC on a standard 2′ silicon wafer (100). After O_2_-plasma activation, the wafer is placed in the DLP printer and the body ② is printed with the UV-curable Formlabs Elastic 50A resin on the mate site of the wafer. The use of the unpolished side improves feature resolution, as it reflects less light than the polished side and allows stronger attachment of the polyacrylate resin to PAC. The pressure applied by the building platform on the tray (approach pressure) as well as the print offset between those two have a significant influence on the print quality. These two parameters require precise adjustment to avoid detachment, squashing or even rupture of the print. A layer-thickness down to 20 μm and a microfluidic channel width as small as 119 μm were reproducibly achieved (see ESI† Fig. S7). The printing of narrower channels was hampered by poor adhesion of cured resin structure to the PAC. To prove the applicability of the process with other acrylate resins, we tested the fabrication process with Pro3dure GR16 (see ESI†). The electrode array was fabricated in parallel, as described in previous works.^33^ Negative photoresist was spin-coated on top of a 2 μm of PAC layer and photolithographically patterned. To improve adhesion the surface was O_2_-plasma-activated, and a 10 nm thick titanium layer, followed by a 100 nm gold layer was deposited, using e-beam physical vapor deposition (PVD). After lift-off and O_2_-plasma-activation, a second 2 μm thick PAC layer was deposited. To allow access to the μ-fluidic component and to the electrodes and their contacts, the top PAC layer was reactive ion etched. The etch pattern was defined by a lithographic process using a positive photoresist. Finally, the conducting polymer (poly(3,4-ethylenedioxythiophene) polystyrene sulfonate (PEDOT:PSS) was ink-jetted onto the electrodes, baked at 120 °C and soaked in deionised water for 12 h. The array was cut and detached from the wafer with water, followed by a manual flip-transfer to a cover slide. After O_2_-plasma-activation the array was aligned, and placed on the printed μ-fluidic layer using a Finetech FINEPLACER pico ma. The heterogeneous integration was then conducted as shown in the ESI.†

### Characterisation of hybrid fabrication and device

The reproducibility of fabrication was tested through several rounds of printing. Six out of eight prints showed sufficient quality.

To quantify the print performance in more detail test specimens were printed. (see ESI† Fig. S8). The failure rate, repeatability with changes in *z*-resolution, total height, feature width and deviation among different prints were evaluated. The planned thickness differs from the measured total thickness by a mean (± standard error of mean) of 18 ± 3 μm for 20 μm *z*-resolution, 20 ± 4 μm for 30 μm *z*-resolution and 7 ± 2 μm for 50 μm *z*-resolution. The measured feature width showing consistently a systematic error from the planned size by a mean (± standard error of mean) of 36 ± 2 μm for the channels and −36 ± 5 μm for the walls. Among different total thickness (300 μm, 200 μm, 150 μm, 100 μm, 50 μm using 50 μm *z*-resolution) the feature width for the 100 μm planned channels and walls deviate by 14 μm and 9 μm. Among different print batches the feature width for the 100 μm planned channels and walls deviate by 6 μm and 10 μm. Using three different z-resolutions (20 μm, 30 μm and 50 μm) the feature width for the 100 μm planned channels and walls deviate by 16 μm and 6 μm. As shown in ESI† Fig. S8 does the failure rate of the features, mainly caused by detaching from the PAC or over exposure, decreased with increasing feature width. The mean surface roughness (root mean square ± standard error of mean) of the bottom PAC amounts to 0.82 ± 0.01 μm, while the one on top of the print amounts to 0.43 ± 0.03 μm. A maximum aspect ratio (H/W) of 2.8 ± 0.3 for channels and 3.4 ± 0.3 for walls and a minimum ratio of 0.37 ± 0.06 for channels and 0.50 ± 0.06 for walls has been achieved.

Accelerated ageing test at 70 °C over 24 h in phosphate-buffered saline (PBS) and 3% aqueous hydrogen peroxide were conducted with PAC-resin-PAC test specimen, reflecting the major features of the implant. No qualitative, visual changes of the test specimen have been noted (see ESI† Fig. S9). Quantitatively, we observed a reduction in mass of 1.6% with PBS and 3.6% with hydrogen peroxide.

The mean burst pressure of the microfluidic channel was measured by custom-made setup (see ESI†). It was found to be 1.1 ± 0.1 bar, confirming that the process used to fabricate the implant provides excellent adhesion between the lithographically fabricated and additively manufactured components (see ESI† Fig. S11). Burst pressure tests conducted on aged implants show similar pressures as the untreated implants, even though a higher total failure rate has been observed. Three out of six of the treated implants were not leaking compared to five out of six for the untreated one. Samples using GR-16 as resin, show a substantially lower burst pressure as elaborated in the ESI.†

To estimate the mechanical properties of the implant, rectangular specimens of the PAC/resin/PAC structure without gold wiring, were fabricated with various resin layer thicknesses and their tensile and flexural moduli were determined using a universal testing machine and custom-made apparatus, described in the ESI.† The Young's modulus was found to be 71 ± 3 MPa (resin layer thickness of 120 μm) 68 ± 5 MPa (165 μm), and 38 ± 2 MPa (240 μm). As expected, these values lie in between the Young's modulus of the resin (3.23 MPa) and that of PAC (4.5 GPa).^32,34^ The flexural modulus was found to be 159 ± 9 MPa (resin layer thickness of 120 μm), 136 ± 18 MPa (165 μm), and 102 ± 9 MPa (240 μm). The measured flexural moduli are larger (*i.e.* the composite is stiffer), than that of pure PAC (4 MPa). Attempts were made to quantify the delamination force by a T-peel-off experiment, but the PAC layer ruptured before any delamination could occur. This finding is in good agreement with the high measured burst pressure and demonstrates the excellent adhesion of the cured resin to the PAC.

Insertion experiments into 0.6% m/v agarose gels which mimic the structural properties of brain tissue showed facile penetration and negligible probe bending for thicknesses above 200 μm (Fig. 2a right). A first attempt to vary the tip's topology by changing the in- and out-of-plane tip angle were successfully conducted (see ESI† Table. S1) and show an impact on the bending within the agarose gel. Hence, investigations of the insertion force are yet inconclusive. Future studies will lay focus on the full utilisation of the additive manufacturing approach to alter the tip shape, potentially reducing the insertion force.

Finally, electrochemical impedance spectroscopy was used to measure electrode impedance. The array was immersed in PBS and a 3 electrode configuration with a Pt counter electrode and a Ag/AgCl reference was used, following published procedures.^35^ The impedance of the PEDOT:PSS electrodes was measured to be 21.2 ± 6.4 kΩ at 1 kHz. Uncoated Au electrodes showed an impedance of 183 kΩ at 1 kHz, confirming that the PEDOT:PSS coating has an impedance-lowering effect.^35^

### *In vivo* validation

To validate the implant's functionality, its ability to interact electrically and chemically with neuronal tissue in the hippocampus of a rat has been investigated. All animal experiments were carried out in accordance with the UK Animals-Scientific Procedures Act (ASPA) 1986. The implant was used to record neural activity in animals under isoflurane anaesthesia (see Fig. 3a and b, in *N* = 3 rats). The implant could also be utilised as a stimulating electrode pair – locally stimulating the tissue electrically. This resulted in interictal-like spikes in the hippocampus, which could also be recorded with the implant (Fig. 3a and b left). Recording and stimulation was carried out using an RHS Stim/Recording system (Intan Technologies), with all data notch-filtered to remove mains noise and band-pass filtered between 1 and 400 Hz.

The drug delivery capabilities of the implant were validated by locally delivering pharmacologically active agents into the hippocampus during electrophysiology recordings. The competitive antagonist MPQX can inhibit neuronal activity and prevent the development of seizures in hippocampal epilepsy models. Electrical stimulation of the naive rat brain, consisting of biphasic 500 μA pulses (1 ms per pulse phase, followed 2 ms of interpulse period) over 300 ms, results in large amplitude interictal-like spikes (Fig. 3a left, ESI† Fig. S16), with a quantified neural activity of 54 ± 14 spikes min^−1^ (Fig. 3a right). Following the administration of MPQX into the hippocampus (54 μM in saline over 30 seconds), electrical stimulation resulted in a significant decrease in interictal spike development (Fig. 3a right) to 14 ± 7 spikes min^−1^ (*p* = 0.014). Further, the convulsant 4AP was delivered into the hippocampus (100 mM in PBS, over 30 seconds), which is known to trigger ictal activity in the hippocampus.^36,37^ An interictal-like events has been triggered successfully and recorded with the implant's electrode array (Fig. 3b), increasingly significantly by a factor of 2.6 (*p* <0.001).

Application of electrical and drug delivery implants, whether in research or clinic, often requires these to remain implanted for long periods of time to perform their role. We therefore characterised the biocompatibility of the implant through implantation into the rat hippocampus and evaluating the astrocytic response 7 days later using GFAP as a marker. The implants triggered an astrocytic response only in the tissue immediately surrounding the implant itself (3.65-fold increase in GFAP intensity, *p* = 0.006, *N* = 4 rats, Fig. 3d).

While GFAP+ astrocytes were enriched in the 20 μm region immediately surrounding the implants, there was no significant enrichment in reactive astrocytes past this thin layer (*p* = 0.106, Fig. 3d). This is indicative of only a very local foreign body reaction, contrasting with the widespread reactive astrocyte response seen in similarly sized stiff silicon implants.^38^ No delamination or degradation were visible in the explanted implants (see ESI† Fig. S15).

In addition to the tissue response, we sought to examine the ability of the multimodal implants and headstage assembly to survive long periods of implantation for chronic studies. The headstage for long-term implantation includes a protective headcap to house the fluidic and electrode lines (Fig. 3e and Fig. S16, ESI†). The implant and headstage were implanted into rats for 7 days, following which their functionality was studied using a similar experimental setup as in Fig. 3a. Of the three animals implanted, MPQX was found to decrease interictal-like activity in only one (Fig. 3f). Upon further investigation of the explanted devices, the fluidic interface between headcap and implant in the remaining two devices (⑨ in Fig. 1) was found to be the point of failure. Further, the formation of a biofilm has been observed (see ESI† Fig S15).

In contrast to this, the electrodes and electrode connections showed good robustness, exhibiting small changes in impedance (1 kHz) from freshly-implanted values (Fig. 3f and g, *p* = 0.58, paired t-test). No electrode failure was observed to occur over the implantation period.

## Discussion

The results presented here show that the combination of additive manufacturing and photolithography can be used to produce multimodal intracranial implants. These hybrid fabricated implants were designed around a hippocampal implantation *in vivo* model using a readily available digital model of the rat anatomy. This ease in design highlights the strength of this technology in producing highly customised devices in a fast and simple way. Inexpensive DLP printing was successfully used to substitute microfabrication of PDMS-based μ-fluidics. The mechanical characterisation and burst test showed that robust, reliable bonding are formed between the PAC and the 3D printed flexible polyacrylate resin. The printing gave good yields with six out of eight printed features being usable for further processing. While the overall hybrid fabrication process is versatile and applicable to various resins, the elastic resin was chosen in this study due to its mechanical properties as well as its adhesiveness in its green state. For the use of other acrylate resins, a variation of exposure time in between the layers is required to achieve a proper bonding to the bottom PAC while retaining a minor cured layer on top. In this study we have exemplified this protocol modification with the Pro3dure GR16 resin.

The sandwich architecture (PAC-resin-PAC) outperforms a two-layered setup (resin-PAC). For latter case the baseplate and walls are printed in one step. We observed the requirement of a minimum thickness of 50 μm to achieve sufficient mechanically stability. Above that the detachment of the print from the building platform is less reliable and lead to damages in the flexible print. The use of a bottom layer of PAC reduces the baseplate to 2 μm and the overall height accordingly.

In an alternative approach based purely around classic microfabrication, the flexible microfluidic layer would be cast and structured first, followed by PAC deposition, and build-up of the electrode array. Here, micro stamping or moulding would have to be used, demanding for a silicon or SU8 master. A redesign of the microfluidic structure would require a redesign of the master, including new mask and new manufacturing process. A further drawback of classic microfabrication approach is the demand for an adhesion promotor between PDMS and PAC.^39^ Without a promotor the two layers are detachable by hand. A bonding of a PAC sheet onto cured PDMS, in an analogous manner to the here reported bonding to a resin, is by best of our knowledge not reported. The here accomplished hybrid fabrication strategy allows not only for a rapid prototyping of the microfluidics with a turnover time of less than an hour, but also a convergent fabrication approach.

Further, with the incorporation of an alignment system into the 3D printer, the presented research might lay the base for inexpensive printing on top of prefabricated features. Overall, our hybrid fabrication approach is a good alternative to soft lithography fabrication.

Importantly, 3D printing was used to implement the electrical and fluidic packaging. Packaging is the unsung hero of bioelectronics, and the approach presented here offers a facile solution to this problem.

From a tissue compatibility perspective, this hybrid fabrication process produces implants with excellent biocompatibility. The use of flexible materials which approach in stiffness biological tissue^2,40^ and of chemically-inert materials, like PAC^41,42^ are both known to contribute to good biocompatibility in implantable devices. While materials used here do not reach the ultra-low stiffness of brain tissue, the incorporation of both soft and flexible materials into the hybrid fabricated implants are likely contributors to the low degree of foreign body reaction observed. The material composite and implant's geometry achieved a good compromise between implantability, as shown by insertion test and low stiffness, to reduce FBR.

In addition to the implant materials themselves, the whole assembly of the hybrid device and electrode and fluidic lines, as well as the protective headcap are robust and can survive chronic implantation in animals for long-term studies. The only exception to this were the mircofluidic headstage-to-implant perpendicular connections, which were found to be prone to failure. Fluidic connections such as these are a well-known point of failure in implants, future work will be aimed for an improved robustness of the fluidic connection to enhance device stability for chronic localised drug delivery applications.

The drop-on-demand PEDOT:PSS coating of the Au electrodes show the expected impedance lowering, even though it limits the achievable size of the electrodes to the minimum size of a droplet (50–30 μm Ø).^43^ The hybrid fabricated implants can effectively record neural activity, electrically stimulate neuronal tissue, and locally deliver compounds in the rat hippocampus. A statistically significant difference between medicated and naïve states proves the success of the localized drug delivery.

## Conclusions

We successfully incorporated 3D printing of a microfluidic system with microfabrication of thin-film microelectrodes into a hybrid fabrication process ideal for small scall production. The commercial elastic resin shows excellent compatibility with the commonly used Parylene-based microelectrode arrays, leading to a robust implant capable of multimodal recording and control of brain function. Future studies will explore the implementation of smaller and higher counts of electrodes, requiring a corresponding adaptation of the used headstage. Further we will focus on utilizing the full potential of the 3D printing in completely replacing the microfabrication part of the process, to achieve a truly rapid prototyping approach. This would lead to a low cost and therefore broadly available technology for biomedical engineering of neuronal implants, enabling an easier translation from academic research to clinical application.

## Author contributions

J. G., T. E. N. and A. C. L. contributed equally to this work. J. G. acquired funding and conceptualized as well as administered the project. He design-engineered and fabricated the implants, contributed to investigation and the corresponding data curation. He visualized the data and wrote the original draft. T. E. N. contributed into investigation, data curation and visualization. A. C. L. acquired funding and participated in the project's conceptualization. He developed the *in vivo* methodology and led their investigation. He visualized the data and wrote the original draft. S. H. contributed to the *in vivo* investigations. R. P. and V. F. C. contributed into the fabrication. V. F. C. established the methodology for microfabrication. D. G. B. supervised and developed the *in vivo* methodology. E. L. K. provided initial resources and participated in funding acquisition and supervision. G. G. M. acquired funding and conceptualized the project. He edited and reviewed the manuscript and supervised the project.

## Conflicts of interest

Asiga and Formlabs participated in consulting manner on this project. No further conflicts are to declare.

## Supplementary Material

MH-009-D1MH01855H-s001
