# Supplementary material for: Hybrid fabrication of multimodal intracranial implants for electrophysiology and local drug delivery
Source: Mater Horiz. 2022 Apr 21;9(6):1727–34. doi: 10.1039/d1mh01855h (PMC9169700; doi:10.1039/d1mh01855h)
Supplement: MH-009-D1MH01855H-s001 [file MH-009-D1MH01855H-s001.pdf]

Electronic Supplementary Material (ESI) for Materials Horizons.
This journal is © The Royal Society of Chemistry 2022

Electronic Supplementary Information for

Hybrid fabrication of multimodal intracranial probes for neural recording and local drug delivery

Johannes Gurke,[†] Tobias E. Naegele,[†] Sam Hilton,a Roberto Pezone, Vincenzo F. Curto, Damiano G. Barone, Emil List-Kratochvil, Alejandro Carnicer-Lombarte,[*] George G. Malliarasa[*]

* Corresponding author: gm603@cam.ac.uk

Content

Methods and Materials 3

Chemicals, Materials and Components 3

General Methods 3

Implant design 4

Fabrication Insights 6

DLP Printing of the electro-fluidic interface 6

DLP Printing on Parylene-C and parameter for Formlabs Elastic FLELCL01[1] 9

DLP Printing on Parylene-C and parameter for Pro3dure GR 16 16

Photolithography and e-beam PVD 19

Photolithography and Reactive Ion Etching 19

Drop-on-demand inkjet printing of custom-made PEDOT-PSS ink 19

Heterogeneous integration of the implant 21

Performance Characteristic of DLP printing on Parylene 22

Accelerated Degradation Test of PAC-RESIN-PAC material composite 25

Mechanical properties 27

Flexural properties 27

Tensile properties 28

Pressure tests 28

Insertion force measurements 29

Electrochemical Impedance Spectroscopy EIS 30

In vivo experiments 31

Animal surgery 31

Electrophysiology recordings and drug delivery 32

Tissue processing and Immunohistochemistry 32

Methods and Materials

Chemicals, Materials and Components

Solvents were purchased from Fisher Scientific and used without further purification. Parylene-C dimer was purchased from Specialty Coating Systems, Inc. Photo resin Young Optics Inc BV-007A and Proc3dure GR 16 Xray for digital light processing 3D printing were purchased from IMakr and Formlabs Elastic FLELCL01 from Simply Rhino Limited and used without further modifications. 2in CZ-Si wafer, thickness = 279 ± 25 µm (100), negative photoresist AZ nLOF 2035 and positive photoresist AZ 10XT 520cP as well as developer AZ 726 MIF by Merck Performance Materials GmbH as well as TechniStrip® NI555 were purchased from MicroChemicals GmbH. Heraeus Clevios™ PH 1000 (PEDOT:PSS) were used. The neuroinhibitor MPQX was purchased from Cambridge Bioscience Limited and 4-aminopyridin (4-AP), glycidyloxypropyl)trimethoxysilane (GOPS), dodecylbenzenesulfonic acid (DBSA) and ethylene glycol (EG) as well as 2-(4-Amidinophenyl)-6-indolecarbamidine dihydrochloride (DAPI), Sodium azide, Phosphate buffered saline and Donkey serum were purchased from Sigma Aldrich and used without further purification. Anti-GFAP and Anti-CD11b+CD11c (OX42) antibody were purchased from abcam. Donkey anti-Rabbit IgG (H+L) highly-absorbed secondary antibody, Alexa Fluor 555 and Donkey anti-Mouse IgG (H+L) highly-absorbed secondary antibody, Alexa Fluor 488 were purchased from ThermoFisher. The Vector® TrueVIEW® Autofluorescence Quenching Kit was bought from Vector Laboratories. Pogo pins (SVPC-P-6269FH01) were produced by N&H Technology GmbH. M0.6x5 mm pan head machine screws (SNZS-S0.6X2-VA) were purchased from MISUMI Europa GmbH. Flex cables (five ways, 1 mm pitch, 68610514422) by Würth Elektronik eiSos GmbH & Co. KG were purchased from RS Components Ltd. 1/32'' Portex Fine Bore Polyethene Tubing by Smiths Medical International Ltd. was purchased from ThermoFisher. High resolution film photomasks were purchased from JD Photodata. High purity oxygen (grade N6.0), sulfur hexafluoride (N3.0), tetrafluoromethane was purchased from BOC.

General Methods

Chemical vapor deposition of PAC was conducted with a Speciality Coating Systems PDS 2010 labcoter 2. Digital light processing (DLP) 3D prints were conducted with an Asiga MAX X UV385 using a for Formlabs Elastic FLELCL01 and Young Optics Inc BV-007A. Filament-based 3D prints were manufactured with an Ultimaker 3. Spin coating was conducted with a Spin coater POLOS 200. Photolithography was conducted with the Karl Suss MA6/BA6 Mask Aligner and e-beam physical vapor deposition (PVD) with an K J Lesker PVD 75 E-beam Evaporator. Reactive ion etching was conducted with a Plasma Pro 80 RIE. For positioning and placing procedures a Fineplacer® Pico ma by FineTech GmbH was used. Optic microscopy was conducted with a Nikon SMZ1270 equipped with a Nikon DFK NME 24UJ003, while for scanning electron microscopy (SEM) a Hitachi TM4000Plus tabletop microscope was used. For washing procedure incl. ultra-

sonication an Bandelin sonorex digiplus DL255H has been used. Dry and tempering procedures were conducted in a Fistreem Vaccum oven equipped with a KNF N840 labport or a Fisherbrand 65K Oven 230V. Surface activations were conducted with a Diener electronics plasma surface technology Zepto RIE equipped with an Agilent IDP-7 Dry Scroll Pump or with a Diener electronics plasma surface technology Femto. Ultraviolet light curing was conducted with an Analytic Jena UVP Crosslinker CL-1000L. Deionized water was received from the water purification system Millipore RiOs -DI® 3UV. For drop-on-demand inkjet printing a Meyer Burger PiXDRO LP50 equipped with a JBinstruments printhead assembly for Fujifilm Dimatrix Cartridges (DMC-11610) were used. Layer thickness measurements were conducted with Dektak XT Profilometer by Brucker. Low level electrical characterisations of circuits were conducted with a Fluke 117 true rms multimeter. For electrochemical impedance spectroscopy a Metrohm Autolab PGSTAT128N was used in a three-electrode configuration, equipped with the Ag/AgCl electrode ZA006 AS RE-1B by ALS Co., Ltd as reference electrode and an Metrohm Platinum-wire electrode as counter electrode. A sinusoidal voltage input of amplitude 10 mV at different frequencies ranging from 1 Hz to 100 kHz was used. All computer aided design were conducted with Autodesk Inventor Pro2020.

Implant design

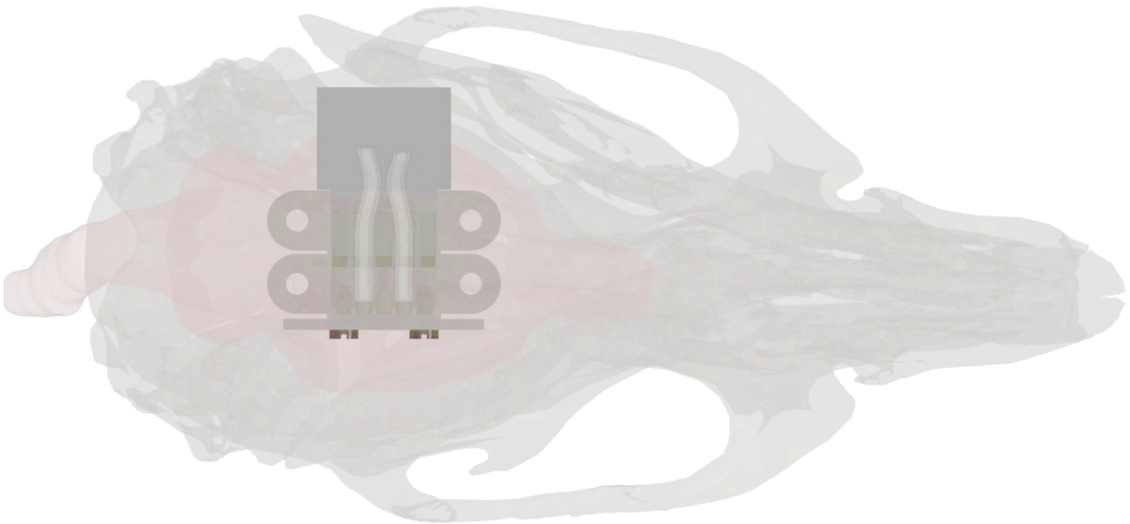

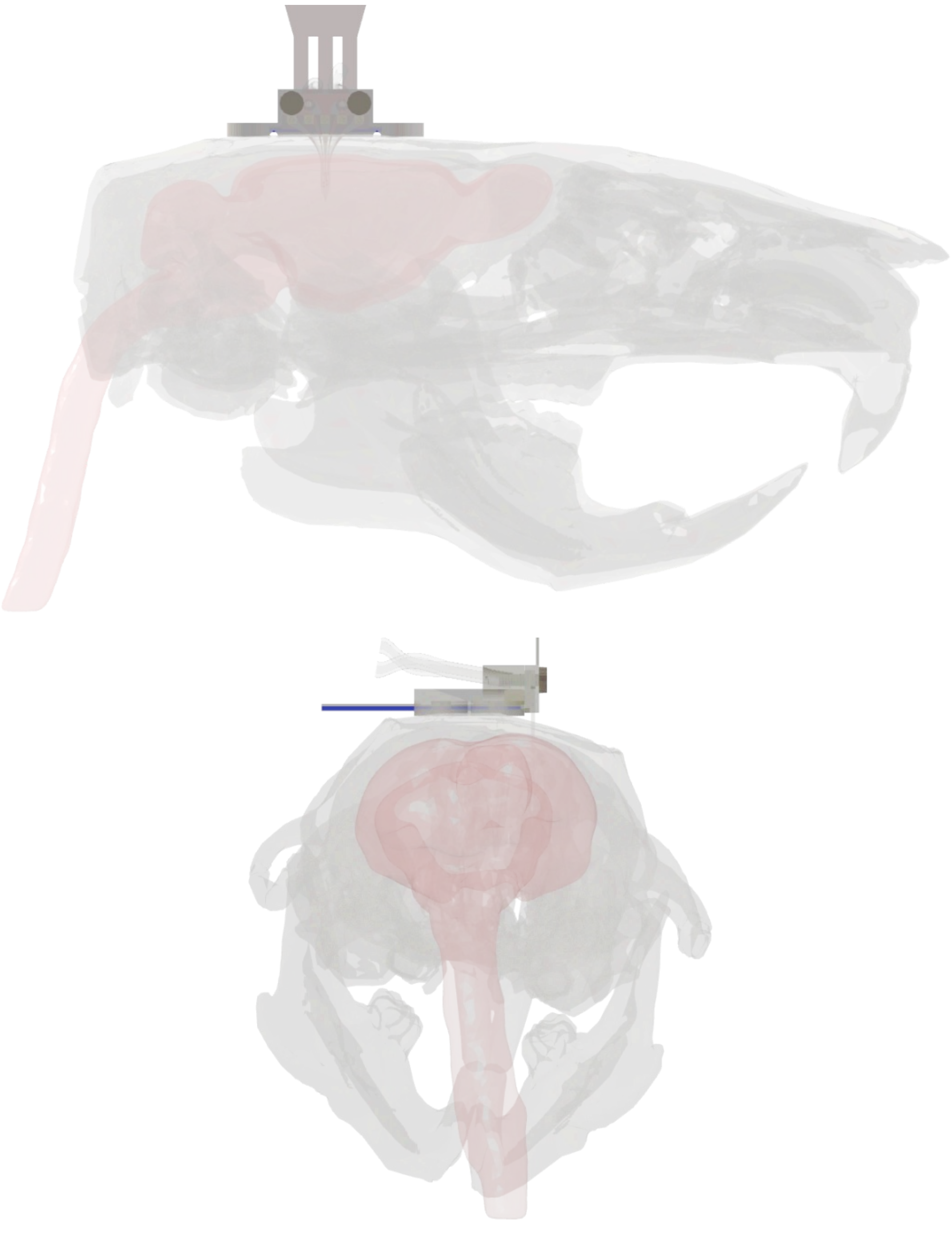

Figure S1 Generic model of a rat brain and skull with implant.[1]

[1]M. P. Bernd, F. Gasca and H. Ulrich G., *IGES and .stl file of a rat brain*, 2013. 1; M. P. Bernd, F. Gasca, O. Christ and H. Ulrich G., *3D .stl file of rat skull*, 2013.

Fabrication Insights

DLP Printing of the electro-fluidic interface

The parts of the interface were printed with Young Optics Inc BV-007A, using the parameter shown below. The prints were sonicated two times in IPA. Afterwards it was allowed to dry under vacuum for 30 min and post-cured (15 min 365 nm)

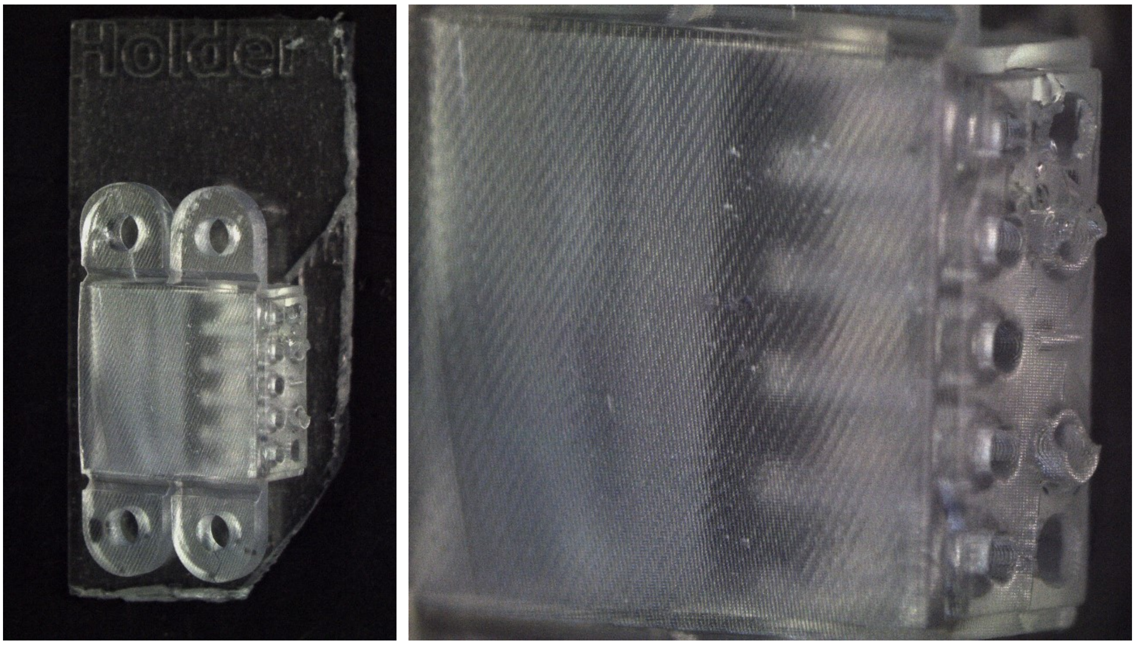

Figure S2 Printed electro-fluidic interface.

Parameter for Young Optics Inc BV-007A [2]

Printer:

IP Address=172.27.10.1
Name=FREEFORM-81D4C2
Type=MAX X UV385
Size=[51.580, 29.014, 76.000] mm

Material:

Name=Young Optics Inc BV-007A_ml_003
Scale=[1.015, 1.015, 1.000] mm

Build Parameters:

Name	Burn-In	1	Units
Print Range From	0.000	0.010	mm
Print Range To	0.010	5.382	mm
Slice Thickness	0.010	0.010	mm
Slice Count	1	537	
Print Range Height	0.010	5.370	mm
Heater Temperature	0.0	0.0	°C
Minimum Temperature	0.0	0.0	°C
Heater Enable	0	0	
Light Intensity	3.00	3.00	mW/cm²
Light Intensity Control	1	1	
Exposure Time	112.000	5.100	s
Z Compensation	0.050	0.050	mm
XY Compensation	0.000	0.000	mm
Support Exposure	300.00	300.00	%
Fill Exposure	100.00	100.00	%
Fill Noise	0.00	0.00	%
Border Exposure	100.00	100.00	%
Border Width	0.000	0.000	mm
Border Noise	0.00	0.00	%
Two-Step Exposure Border Width	0.000	0.000	mm
Separation Velocity	2.4750	2.4750	mm/s

[2] Provided by Asiga and further optemized.

Separation Acceleration	0.000	0.000	mm/s²
Separation Deceleration	0.000	0.000	mm/s²
Separation Distance	10.000	10.000	mm

Name	Burn-In	1	Units
Separation Detect Window	15	15	g
Separation Detect Window Time	0.350	0.350	s
Separation Detect Hard Stop	1	1	
Separation Pressure Limit	300.000	300.000	g/cm²
Approach Velocity	4.3000	4.3000	mm/s
Approach Acceleration	0.000	0.000	mm/s²
Approach Deceleration	0.000	0.000	mm/s²
Approach Pressure Limit	100.000	100.000	g/cm²
Tare Interval	0.001	0.001	mm
Pressure Hysteresis	5.00	5.00	%
Layer Tolerance	30.00	30.00	%
Viscosity Range	1.000	1.000	mm
Motor Timeout	150.000	150.000	s
Traverse Timeout Range	0.300	0.300	mm
Wait Time (After Exposure)	5.000	0.500	s
Wait Time (After Separation)	5.000	0.000	s
Wait Time (After Approach)	0.000	0.000	s

DLP Printing on Parylene-C and parameter for Formlabs Elastic FLELCL01[1]

A 2in silicon wafer is coated with 2µm PAC and activated *via* a O_2 plasma (60 s, 0.25 mbar, 100 W, 40 kHz). The wafer placed on the building platform with a custom-designed alignment tool (ultimaker 3, see .stl files), and attached to the building platform by double-sided adhesive tape. Using the parameters shown below, the resin was printed on the PAC-coated wafer. After printing excess of uncured resin was removed with an air-blow gun. The prints were washed three times, each with IPA in a low power sonication (max 2min in total, 40%), to avoid detachment of the print from the PAC. After washing the print was dried with an air-blow gun and then for 30min at room temperature. (Yield 75 %, N = 5)

Parameter for Formlabs Elastic FLELCL01

Printer Parameter:

IP
Address=172.27
.10.1
Name=FREEFOR
M-81D4C2
Type=MAX X
UV385
Size=[51.580, 29.014, 76.000] mm

Material:

Name=Formlabs Elastic
FLELCL01_002_SW Scale=[1.000,
1.000, 1.000] mm

Build Parameters:

Name	Burn-In	1	2	Units
Print Range From	0.378	0.388	0.428	mm
Print Range To	0.388	0.428	0.678	mm
Slice Thickness	0.010	0.040	0.050	mm
Slice Count	1	1	5	
Print Range Height	0.010	0.040	0.250	mm
Heater Temperature	35.0	35.0	35.0	°C
Minimum Temperature	33.0	33.0	33.0	°C
Heater Enable	1	1	1	
Light Intensity	29.33	29.33	29.33	mW/cm²
Light Intensity Control	0	0	0	
Exposure Time	0.640	0.800	0.880	s
Z Compensation	0.120	0.120	0.120	mm
XY Compensation	-0.015	-0.015	-0.015	mm
Support Exposure	100.00	100.00	100.00	%
Fill Exposure	100.00	100.00	100.00	%
Fill Noise	0.00	0.00	0.00	%
Border Exposure	100.00	100.00	100.00	%

Border Width	0.000	0.000	0.000	mm
Border Noise	0.00	0.00	0.00	%
Two-Step Exposure Border Width	0.000	0.000	0.000	mm
Separation Velocity	2.4750	2.4750	2.4750	mm/s
Separation Acceleration	0.000	0.000	0.000	mm/s^2
Separation Deceleration	0.000	0.000	0.000	mm/s^2
Separation Distance	10.000	10.000	10.000	mm
**Name**	**Burn-In**	**1**	**2**	**Units**
Separation Detect Window	0	0	0	g
Separation Detect Window Time	0.000	0.000	0.000	s
Separation Detect Hard Stop	1	1	1	
Separation Pressure Limit	300.000	300.000	300.000	g/cm^2
Approach Velocity	0.1525	0.1525	0.1525	mm/s
Approach Acceleration	0.000	0.000	0.000	mm/s^2
Approach Deceleration	0.000	0.000	0.000	mm/s^2
Approach Pressure Limit	20.000	19.000	19.000	g/cm^2
Tare Interval	0.001	0.001	0.001	mm
Pressure Hysteresis	5.00	5.00	5.00	%
Layer Tolerance	30.00	30.00	30.00	%
Viscosity Range	1.000	1.000	1.000	mm
Motor Timeout	150.000	150.000	150.000	s
Traverse Timeout Range	0.300	0.300	0.300	mm
Wait Time (After Exposure)	2.000	0.100	0.100	s
Wait Time (After Separation)	5.000	0.000	0.000	s
Wait Time (After Approach)	0.000	0.000	0.000	s

Table S1 Various tip shapes.			
Topo<br>-logy	3 Edges	4 Edges	5 Edges
10°<br>(12.1<br>±<br>0.2°)	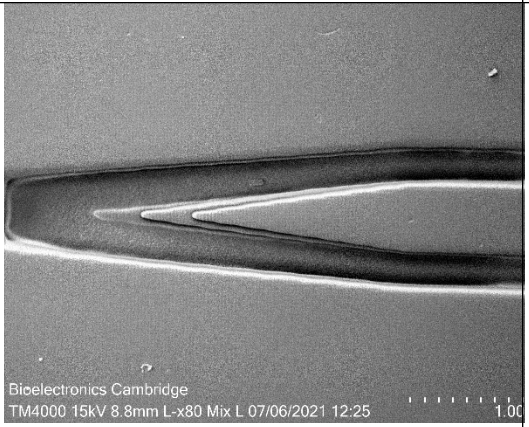	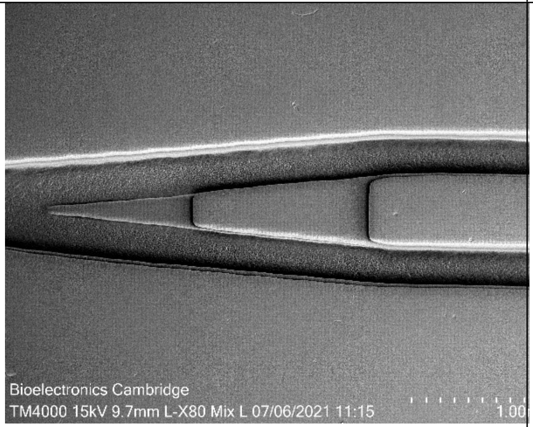	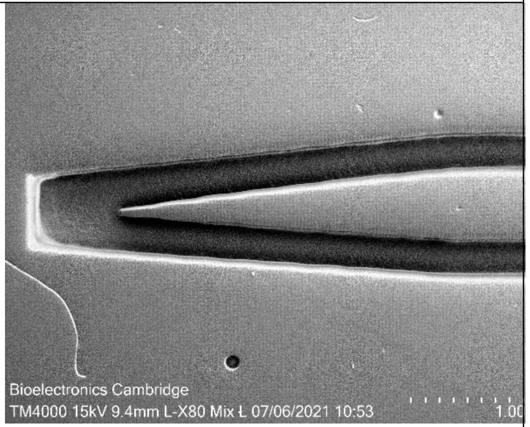
20°<br>(24.2<br>±<br>0.1°)	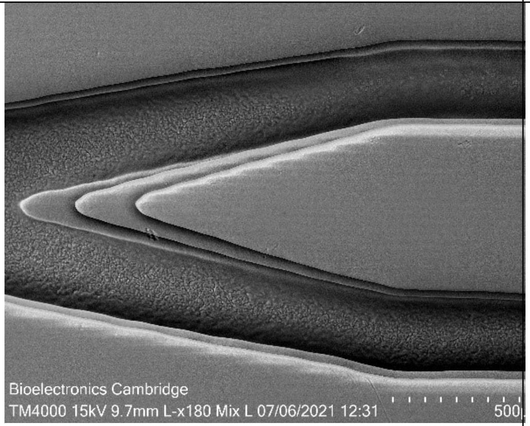	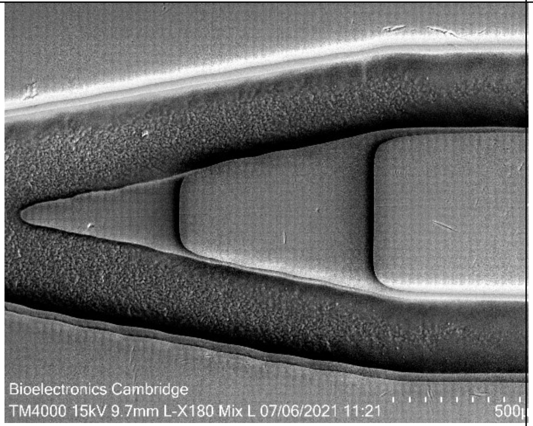	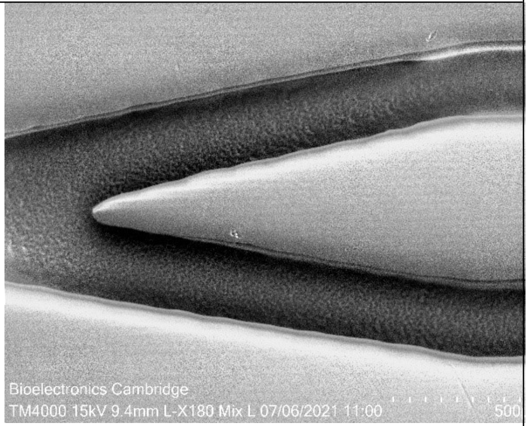

<table>
<tr>
<td>30°
(35.1
±
1.7°)</td>
<td>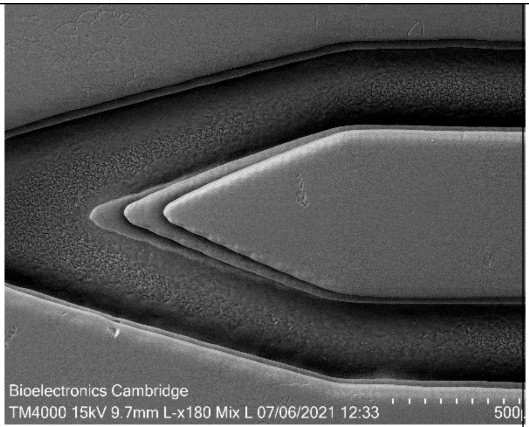
</td>
<td>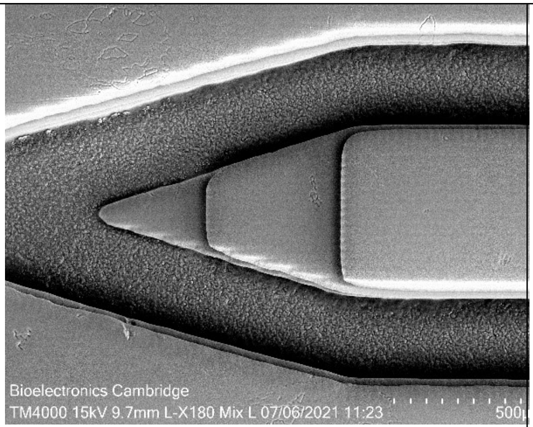
</td>
<td>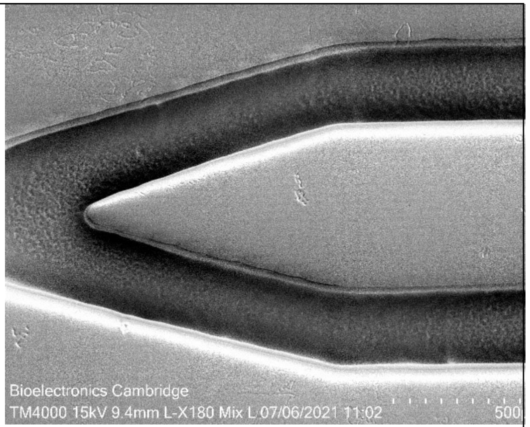
</td>
</tr>
<tr>
<td>40°
(44.9
±
2.8°)</td>
<td>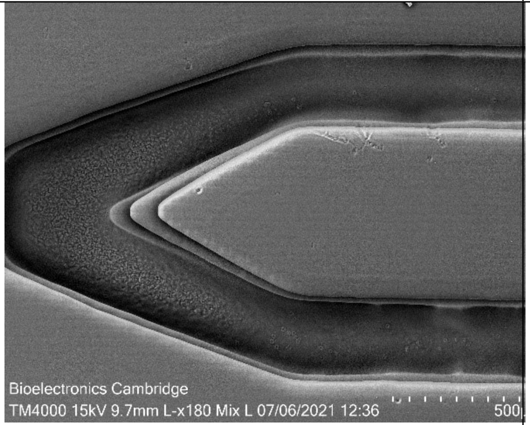
</td>
<td>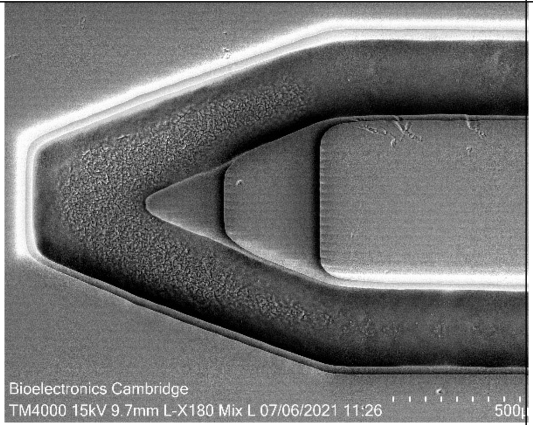
</td>
<td>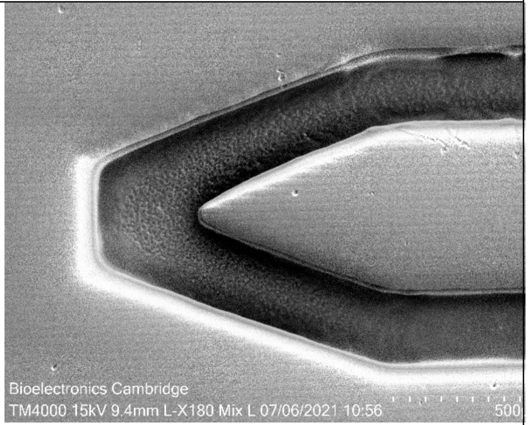
</td>
</tr>
</table>

<table>
<tr>
<td>50°
(56.8
±
2.6°)</td>
<td>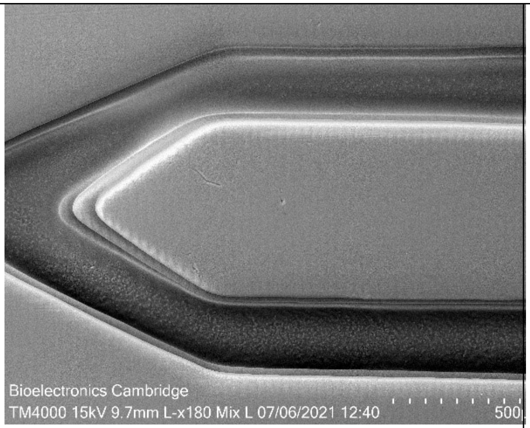
</td>
<td>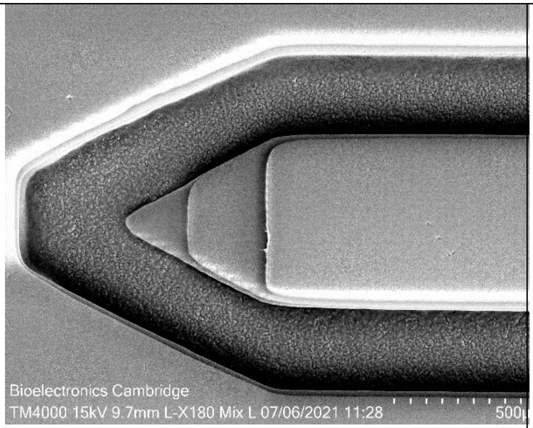
</td>
<td>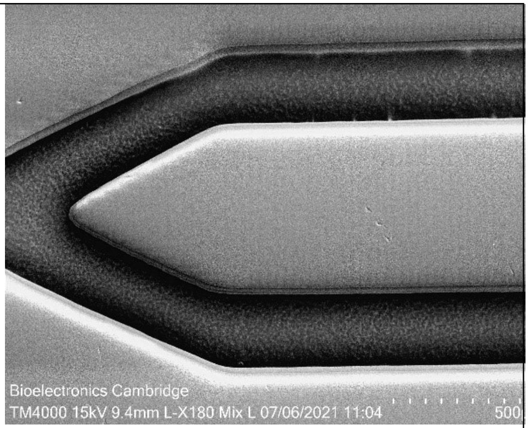
</td>
</tr>
</table>

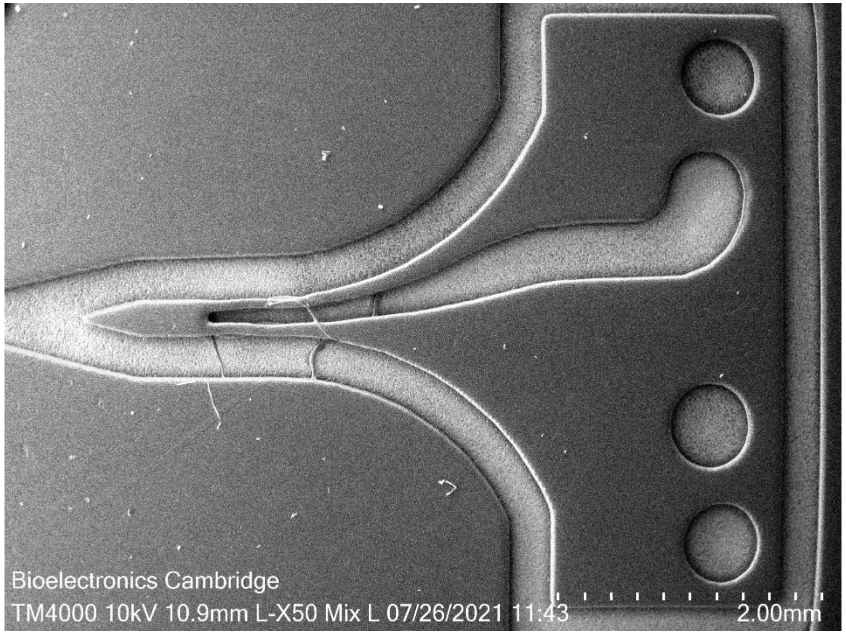

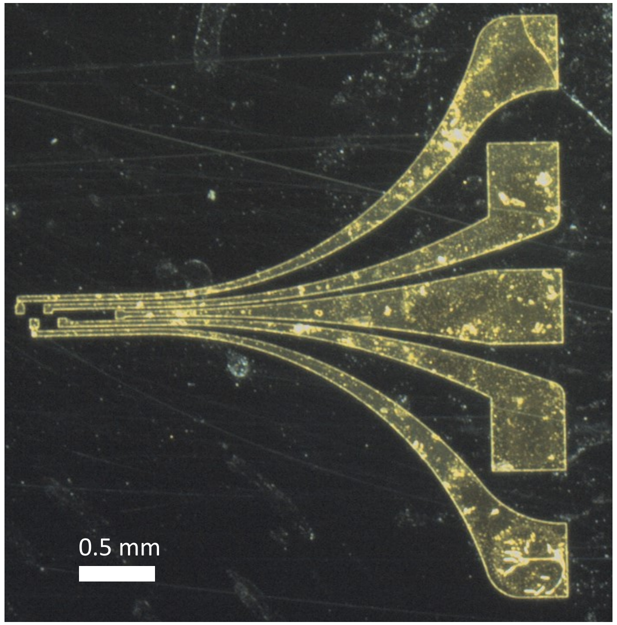

Figure S3 SEM Picture of a one channel μ-fluidic system and dimentinal test.

DLP Printing on Parylene-C and parameter for Pro3dure GR 16

A 2in silicon wafer is coated with 2µm PAC and activated *via* a O_2 plasma (60 s, 0.25 mbar, 100 W, 40 kHz). The wafer placed on the building platform with a custom-designed alignment tool (ultimaker 3, see .stl files), and attached to the building platform by double-sided adhesive tape. Using the parameters shown below, the resin was printed on the PAC-coated wafer. After printing excess of uncured resin was removed with an air-blow gun. The prints were washed three times, with IPA and DIW in a low power sonication (max 2min in total, 40%), to avoid detachment of the print from the PAC. After washing the print was dried with an air-blow gun and then for 30min at room temperature. (Yield 37.5 %)

Printer Parameter:

IP Address=172.27.10.1
Name=FREEFORM-81D4C2
Type=MAX X UV385
Size=[51.580, 29.014, 76.000] mm

Material:

Name=printodent GR-16 Xray D1001401 - 385Scale=[1.000, 1.000, 1.000] mm

Build Parameters:

Name	Burn-In	1	2	Units
Print Range From	0.385	0.400	0.550	mm
Print Range To	0.400	0.550	0.565	mm
Slice Thickness	0.015	0.030	0.015	mm
Slice Count	1	5	1	
Print Range Height	0.015	0.150	0.015	mm
Heater Temperature	35.0	35.0	35.0	°C
Minimum Temperature	34.5	34.5	34.5	°C
Heater Enable	1	1	1	
Light Intensity	6.00	6.00	6.00	mW/cm²
Light Intensity Control	1	1	1	
Exposure Time	1.000	0.825	0.700	s
Z Compensation	0.000	0.000	0.000	mm
XY Compensation	0.000	0.000	0.000	mm
Support Exposure	100.00	100.00	100.00	%
Fill Exposure	100.00	100.00	100.00	%
Fill Noise	0.00	0.00	0.00	%

Border Exposure	100.00	100.00	100.00	%
Border Width	0.000	0.000	0.000	mm
Border Noise	0.00	0.00	0.00	%
Two-Step Exposure Border Width	0.000	0.000	0.000	mm
Separation Velocity	2.4750	2.4750	2.4750	mm/s
Separation Acceleration	0.000	0.000	0.000	mm/s^2
Separation Deceleration	0.000	0.000	0.000	mm/s^2
Separation Distance	10.000	10.000	10.000	mm

Name	Burn-In	1	2	Units
Separation Detect Window	0	0	0	g
Separation Detect Window Time	0.000	0.000	0.000	s
Separation Detect Hard Stop	1	1	1	
Separation Pressure Limit	100.000	100.000	100.000	g/cm²
Approach Velocity	0.1525	0.1525	0.1525	mm/s
Approach Acceleration	0.000	0.000	0.000	mm/s²
Approach Deceleration	0.000	0.000	0.000	mm/s²
Approach Pressure Limit	100.000	50.000	50.000	g/cm²
Tare Interval	0.001	0.001	0.001	mm
Pressure Hysteresis	5.00	5.00	5.00	%
Layer Tolerance	30.00	30.00	30.00	%
Viscosity Range	1.000	1.000	1.000	mm
Motor Timeout	300.000	300.000	300.000	s
Traverse Timeout Range	0.100	0.100	0.100	mm
Wait Time (After Exposure)	0.000	0.000	0.000	s
Wait Time (After Separation)	0.000	0.000	0.000	s
Wait Time (After Approach)	0.000	0.000	0.000	s

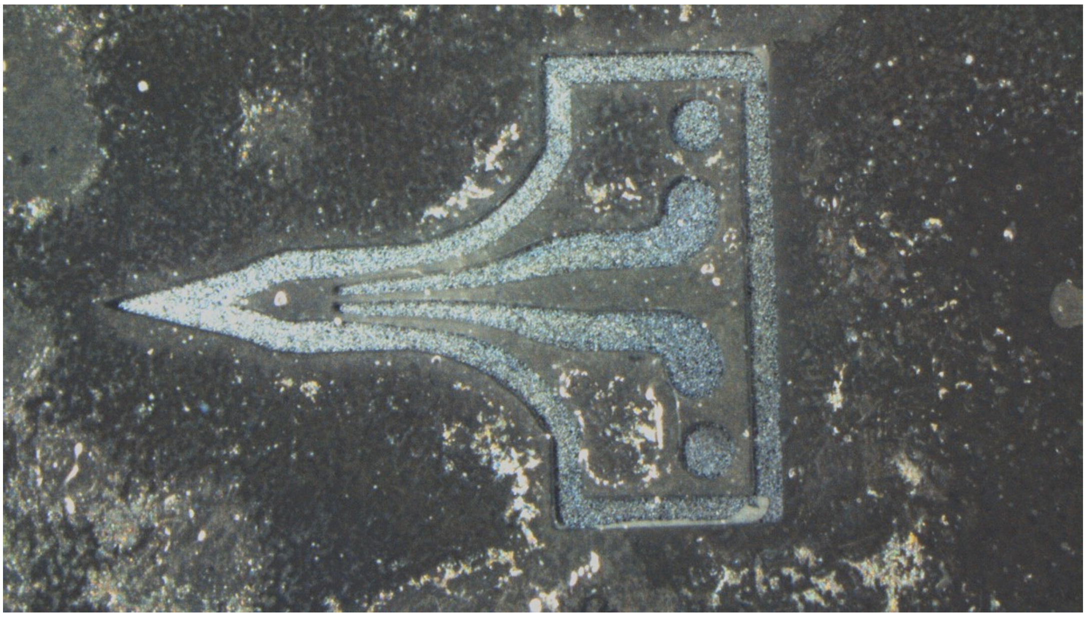

Figure S4 Print with Pro3dure GR-16

Photolithography and e-beam PVD

AZ nLof 2035 was spin-coated on a PAC coated 2in wafer (500 rpm, 1000 rpm/s for 5s; 3000 rpm, 8000 rpm/s for 30s), followed by a soft baking step for 60s at 110°C on a hot plate. UV-Exposure were conducted with a mask aligner in one step of 160 mJ/cm^2. Post exposure baking were conducted at 108°C for 80 s, followed by developing with AZ726 MIF for 12-16 s, followed by rinsing with DI water and air blow drying. The samples were activated with an O_2 plasma (60 s, 0.4 mbar, 100 W, 40 kHz) before ebeam PVD. First 10 nm titanium with a rate of 0.03 nm/s was deposited, followed by 100 nm gold with a rate of 0.05 nm/s. The lift off were conducted in Ni 555 stripper for 24 h, followed by rinsing with DI water, acetone and IPA, as well as gentle wiping, if required. Finally, the samples were air blow dried, O_2 plasma activated (60 s, 0.4 mbar, 100 W, 40 kHz) and coated with 2 µm PAC.

Photolithography and Reactive Ion Etching[3]

The photoresist AZ 10XT was spin-coated on the samples (500 rpm, 1000 rpm/s for 5s, 3000 rpm, 8000 rpm/s for 30s), followed by soft baking for120 s at 110°C and 30 min post backing delay. The UV-exposures were conducted in two steps of 15 s (17-20 mW/cm^2) with a wait time of 5 s in between the exposure steps. The samples were developed in AZ 726 MIF for 5 min, followed by rinsing DI water. The reactive ion etching was conducted in a mixture of perfluoro methane, sulfur hexafluoride and oxygene (60 mTorr, 180 W, 8.0 sccm CF_4, 2.0 sccm SF_6, 50.0 sccm O_2).

Drop-on-demand inkjet printing of custom-made PEDOT-PSS ink[4]

First, 30 mg (91.8 µmol, 15.3 mM) DBSA were dissolved in1.25 mL PEDOT:PSS dispersion, 1.25 mL DIW, and 62.5 µL (5 vol% of PEDOT:PSS) ethylene glycol by shaking and sonication for 15 min. Right before use, 11.6 µL (12.4 mg, 52.5 µmol) GOPS were added, and the mixture were shaken gently, followed by filtration through a 0.8 um PTFE filter into DMC cartridge. A high-resolution bitmap of the print pattern was generated using Adobe Illustrator and Photoshop. The samples were activated with an O_2 plasma (60 s, 0.25 mbar, 100 W, 40 kHz) before inkjet printing. The ink was jetted at 1000 Hz firing frequency with a wave consisting of an up down phase of 0µs, idle before firing of 0 µs, fill ramp of 0.4 µs, time low of 3.5 µs, fire ramp of 2.1 µs, time high 3.3 µs, end ramp 0.3 µs, idle after firing of 2.9 µm, utilizing a pulse shape of 2 V minimum, 10 V medium and 25-27 V high potential The pressure setpoint were adjusted to -2.0 mbar. Cartridge and table temperature were set to 30°C. Nozzle purge as well as jetting and drop shape analysis were conducted before every print. Afterwards, the samples were dried at 80°C under vacuum, to remove access water for 20 min, followed by hard backing at 135°C for 60 min. The film was stored in distilled water for 12 h to remove excess PSS.

[3] J. A. DeFranco, B. S. Schmidt, M. Lipson and G. G. Malliaras, *Organic Electronics*, 2006, **7**, 22-28.
[4] Rivnay J, Inal S, Collins B A, Sessolo M, Stavrinidou E, Strakosas X, Tassone C, Delongchamp D M and Malliaras G G *Nat. Commun.* 2016 **7** 11287.

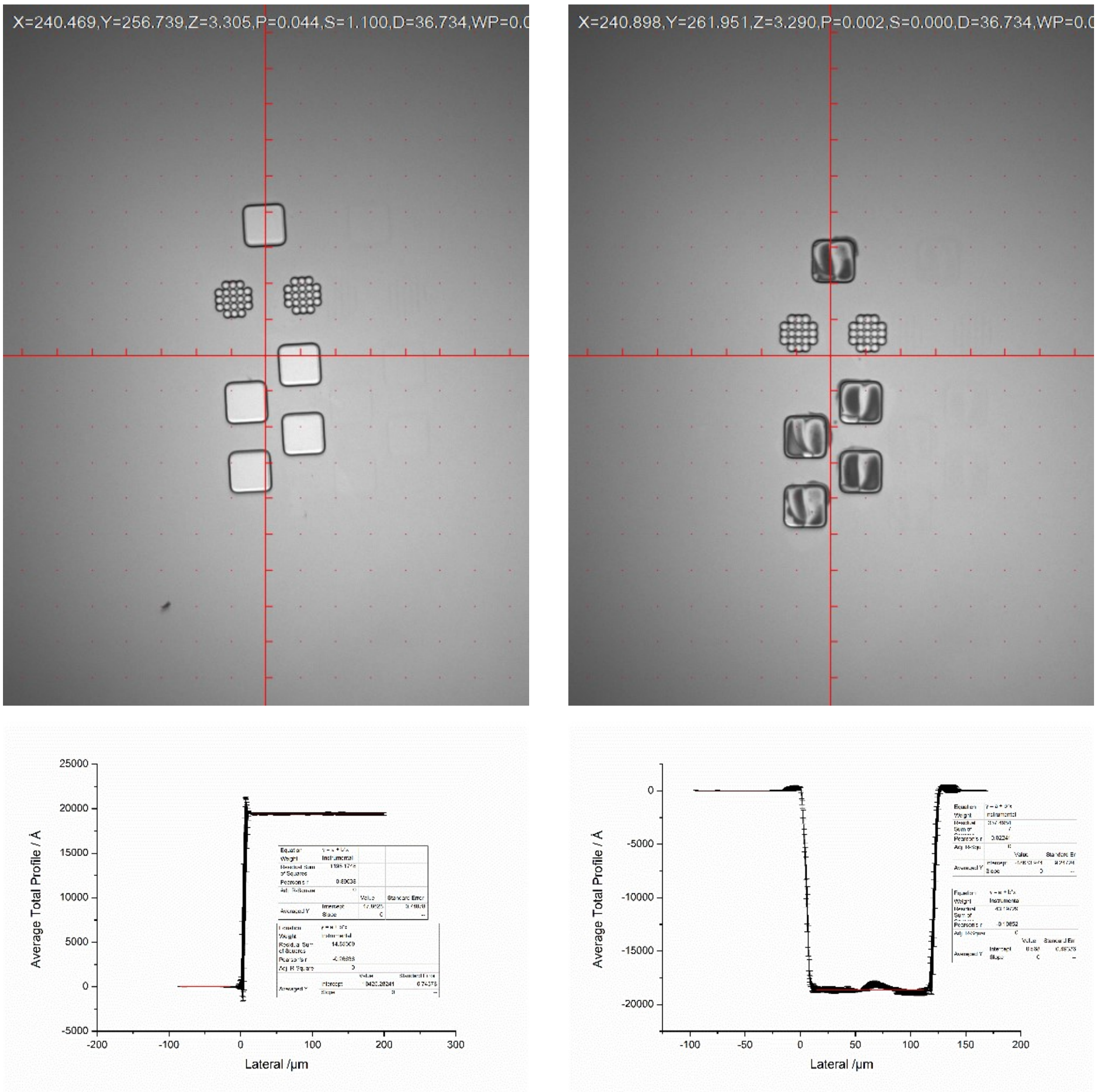

Figure S5 Drop-on-demand ink jetting on dummies. top left: Etched PAC on Si-Wafer; top right: Drop-on-demand ink jetted PEDOT:PSS onto etching for electrode pads; bottom left: Profilometry of the uncoated electrode pads (N=2); bottom right: Profilometry of the uncoated electrode pads (N=5), giving 77 ± 11 nm layer thickness.

Heterogeneous integration of the implant for acute in vivo experiments

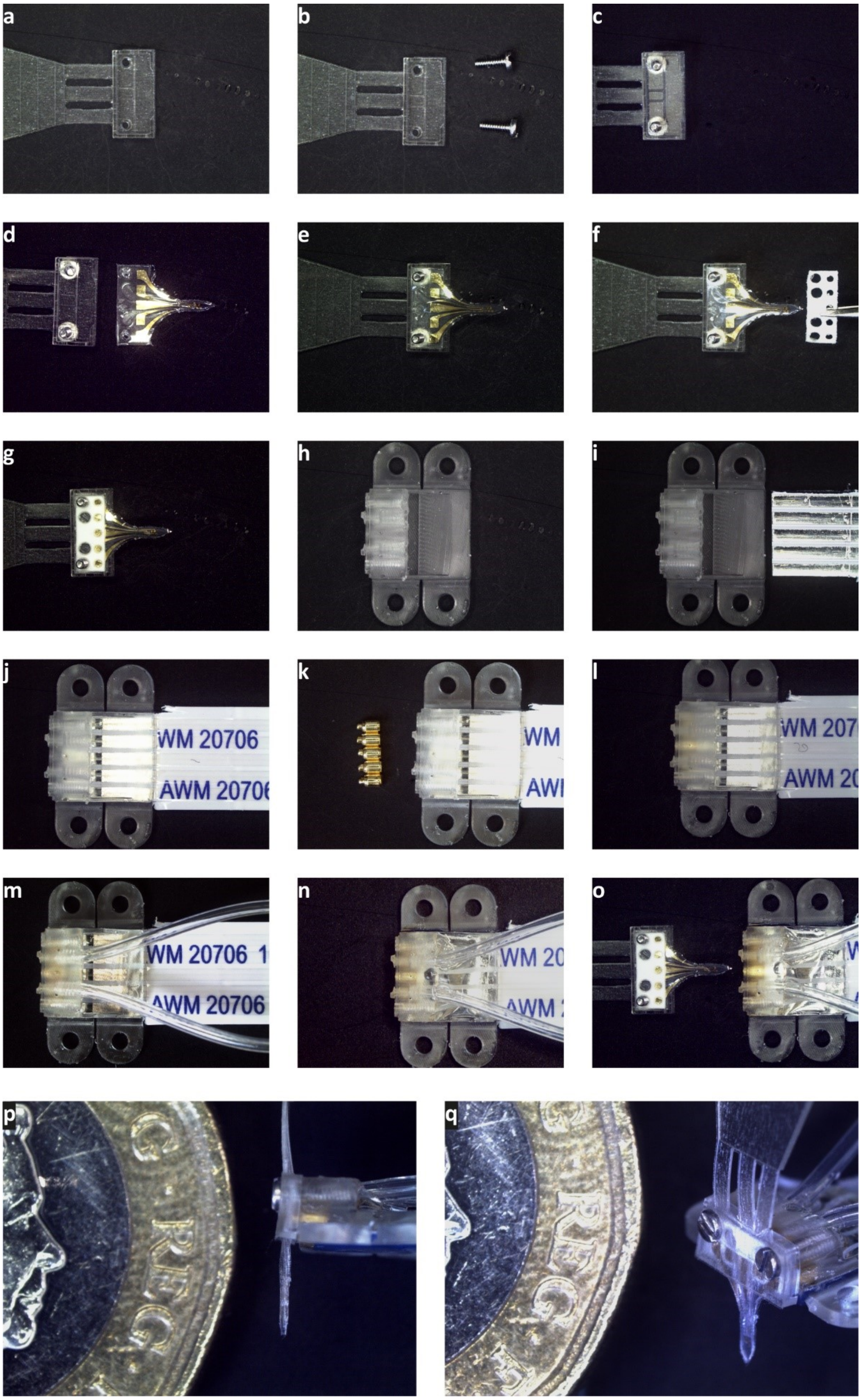

Figure S6 Heterogeneous integration of the implant: **a** 3D printed socket; **b** and **c** assembly of M0.6 machine screws into socket; **d**-**e** assembly of the hybrid fabricated probe; **f** and **g** assembly of the double-sided adhesive tape; **h** 3D printed electro-fluidic interface; **i** and **j** insertion of the flex cable; **k** and **l** insertion of the pogo pins; **m** and **n** insertion of 1/32’’ polyethylene tubing and encapsulation with low-viscosity UV curable resin(BV-007); **o** merging of interface with probe; **p** side view and **q** perspective view of the assembly with 1 pound sterling coin for scale.

Heterogeneous integration of the implant for long term in vivo experiments

For long term the socket has been exchanged. Here it is made from a tough, yet biocompatible resin (free form temp) to it withstand freely moving rates. The 3D printed socket allows the easy implantation, wire and tube management as well as a mounting of a standardized headcap with lid. The M0.6 fasteners were here replaced by a twisted wire, achieving a compressing force.

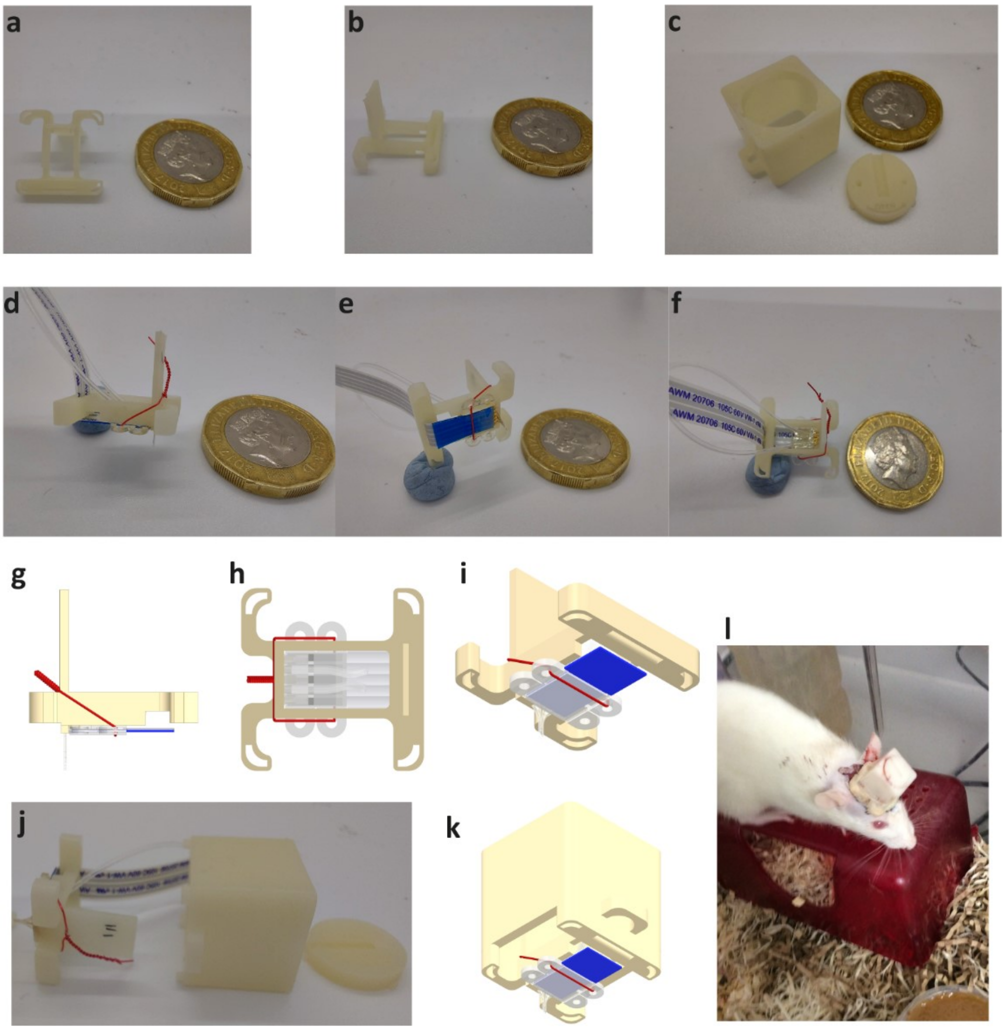

Figure S7 Implant for long-term validation: **a** and **b** 3D printed socket; **c** headcap with lid; **d-i** assembly of the hybrid fabricated probe; **j-l** 3D printed assembly of the headstage.

Performance Characteristic of DLP printing on Parylene

In order to quantify the performance (i.e. reliability, repeatability, surface roughness and aspect ratio) of the DLP printing process on parylene C, test specimens were printed. The test patterns consist of three walled channels with designed widths of 80 µm, 100 µm, 150 µm, 200 µm and 300 µm. Fig SX left, top illustrates the test pattern. Four test specimens using a z-resolution (i.e. 3D print layer thickness) of 50 µm, one specimen with a layer thickness of 20 µm and one with 30 µm were produced. The 50 µm z resolution sample holds has features with 50 µm, 100 µm, 150 µm, 200 µm and 300 µm total thickness. The specimen with a z resolution of 20 µm has patterns of 40 µm, 60 µm, 80 µm, 100 µm, 200 µm, and 300 µm thickness and the sample with a z resolution of 30 µm has features with 60 µm, 150 µm and 300 µm total thickness. The test specimens were characterised using scanning electron microscopy (SEM) and profilometry. The width of the features was determined by SEM imaging the samples and analysing the data using the image processing program ImageJ (National Institutes of Health), measuring each feature three time. The failure rate was calculated by counting the printed features (N=60 for every planned feature width). A successful channel print must allow fluid flow and the bottom PAC must be visible. A successful wall print show no or just minor detachment from the PAC, overcuring or disalignment of the layers. We expected an isotropic printing in XY direction, hence the rotation around the Z-axis was not part of the evaluation. Both a variance analysis of the thickness and feature width were conducted.

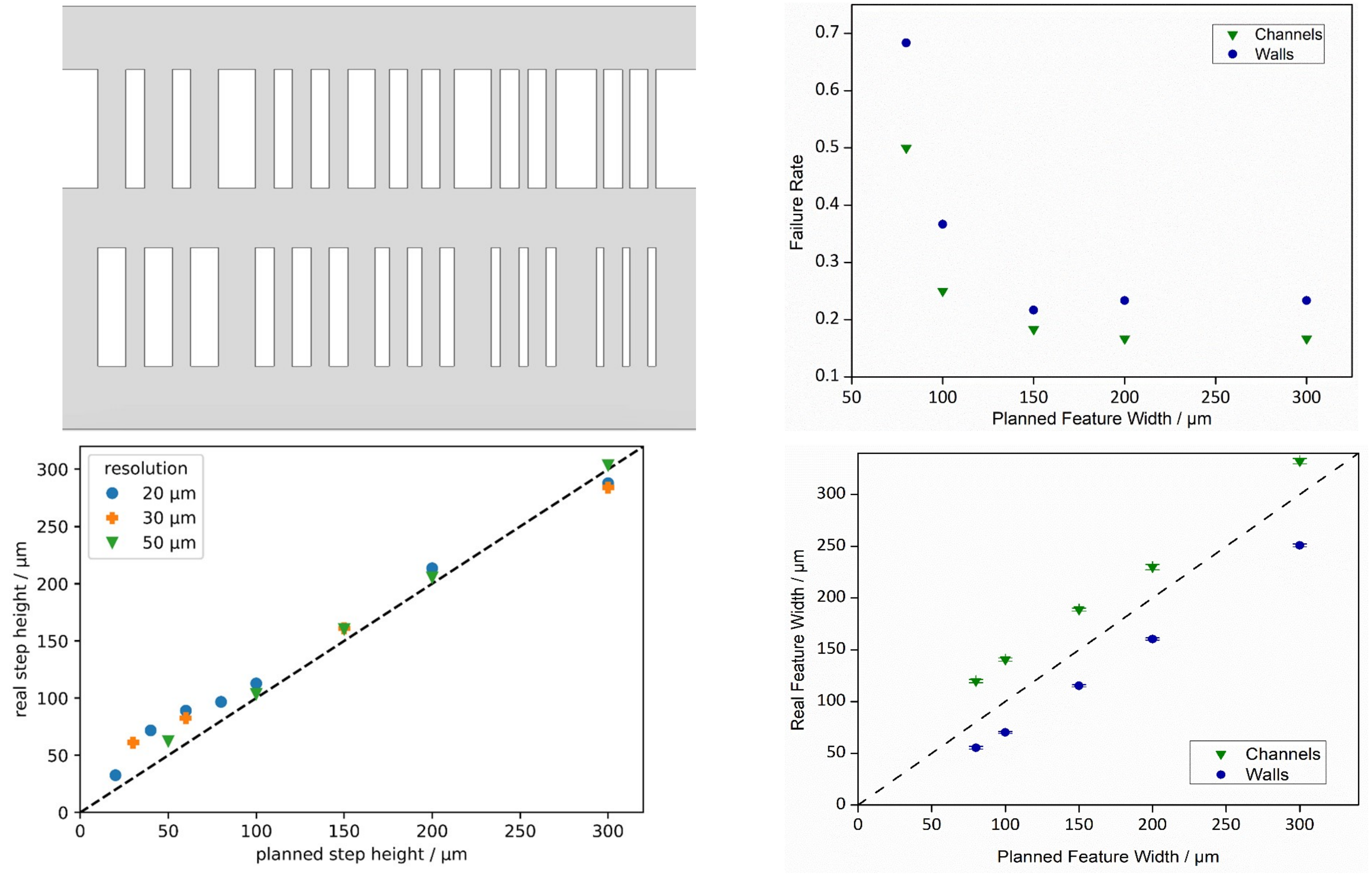

Figure S8 Characterising the printing performance: Top left: test pattern holding from left to right features with 300 µm, 200 µm, 150 µm, 100 µm and 50 µm width; top left failure rate with N=60 each; plot of the planned vs the measured total thickness using three z-resolution (20 µm, 30 µm and 50 µm)[5]; plot of the planned vs the measured feature width using 50 µm z-resolution.

[5] The individual errors are all smaller 3.5um and have been omitted in this figure to improve readability.

The planed thickness differs from the measured total thickness by a mean (± standard error of mean) of 18 ± 3 µm for 20 µm z-resolution, 20 ± 4 µm for 30 µm z-resolution and 7 ± 2 µm for 50 µm z-resolution. The measured feature width showing consistently a systematic error from the planed size by a mean (± standard error of mean) of 36 ± 2 µm for the channels and -36 ± 5 µm for the walls. Among different total thickness (300 µm, 200 µm, 150 µm, 100 µm, 50 µm using 50 µm z-resolution) the feature width for the 100 µm planed channels and walls deviate by 14 µm and 9 µm. Among four different wafers the feature width for the 100 µm planned channels and walls deviate by 6 µm and 10 µm Using three different z-resolutions (20 µm, 30 µm and 50 µm) the feature width for the 100 µm planned channels and walls deviate by 16 µm and 6 µm. (standard deviation). As shown in Fig S7 does the failure rate of the features, mainly caused by detaching from the PAC or over exposure, decrease with increasing feature width. The mean surface roughness (root mean square) of the bottom PAC amounts to 0.82 ± 0.01 µm, while the one on top of the print amounts to 0.43 ± 0.03 µm. A maximum aspect ratio (H/W) of 2.8 ± 0.3 for channels and 3.4 ± 0.3 for walls and a minimum ratio of 0.37 ± 0.06 for channels and 0.50 ± 0.06 for walls has been achieved.

Accelerated Degradation Test of PAC-RESIN-PAC material composite[6]

The degradation test was conducted using a comb-like specimen, where every comb tooth is 500 µm thick, similar to the width of the implant. The specimen was prepared as shown in fabrication schema in the main manuscript. The degradation was performed with PBS and 3 % aqueous hydrogen peroxide solution. The test specimens were dried in vacuum at 80 °C to constant mass and weighted with an analytical balance (OHAUS Pioneer Plus analytical PA224C), giving minimum wight of 20 µm, a readability and repeatability of both 0.1 mg. The samples were immersed in 1.5 mL PBS or 3 % aqueous hydrogen peroxide solution, respectively, and shook for 24 h at 70 ± 2 °C. Afterwards the solution was decanted and washed twice with 1.5 DIW. The test specimens were dried in vacuum at 80 °C to constant mass. The test results are shown in Table S1. Qualitatively no changes have been observed.

Table S2 Summery Print performance

**Failure Rate**				
**Planned Feature Width /mm**	**Count of successful channels (n = 60)**	**Failure Rate**	**Count of successful walls (n = 60)**	**Failure Rate**

[6] ISO 10993-13

0.08	30	0.5	19	0.68333
0.1	45	0.25	38	0.36667
0.15	49	0.18333	47	0.21667
0.2	50	0.16667	46	0.23333
0.3	50	0.16667	46	0.23333

Feature consistency among different print batches

**Wafer No**	**Mean Channel Width / mm**	**SE of mean/mm**	**Mean Wall Width /mm**	**SE of mean/mm**
1	0.15946	0.00347	0.0707	0.00153
2	0.1474	0.00115	0.05547	0.00121
3	0.12587	0.00304	0.07602	0.00118
4	0.13781	0.00189	0.06628	0.00263

Feature consistency among different total thicknesses

**Planned Total Thickness /µm**	**Mean Channel Width / mm**	**SE of mean/mm**	**Mean Wall Width /mm**	**SE of mean/mm**
300	0.13893	0.00395	0.08833	0.0016
200	0.13617	0.00267	0.07033	0.00197
150	0.13288	0.00217	0.06947	0.00154
100	0.14453	0.00184	0.06931	0.00135
50	0.14708	0.00454	0.06286	0.00158

Feature consistency among different Z-Resolutions

Z-Resolution / µm	Channel Width / mm	SE of Mean / mm	**Wall Width / mm**	**SE of Mean /mm**
20	0.11347	0.00186	0.0739	0.00306
30	0.14028	0.00257	0.06211	0.00462
50	0.14057	0.00154	0.07025	9.23701E-4

Aspect Ratio

	**Measured Total Thickness /µm**	**Measured Feature Width /µm**	**H/W**
**Channel**	32.5 ± 2.8	87.2 ± 5.8	0.37 ± 0.06

**Wall**	32.5 ± 2.8	64.9 ± 2.8	0.50 ± 0.06
**Channel**	303.5 ± 1.4	108.2 ± 10.9	2.8 ± 0.3
**Wall**	303.5 ± 1.4	88.3 ± 6.8	3.4 ± 0.3

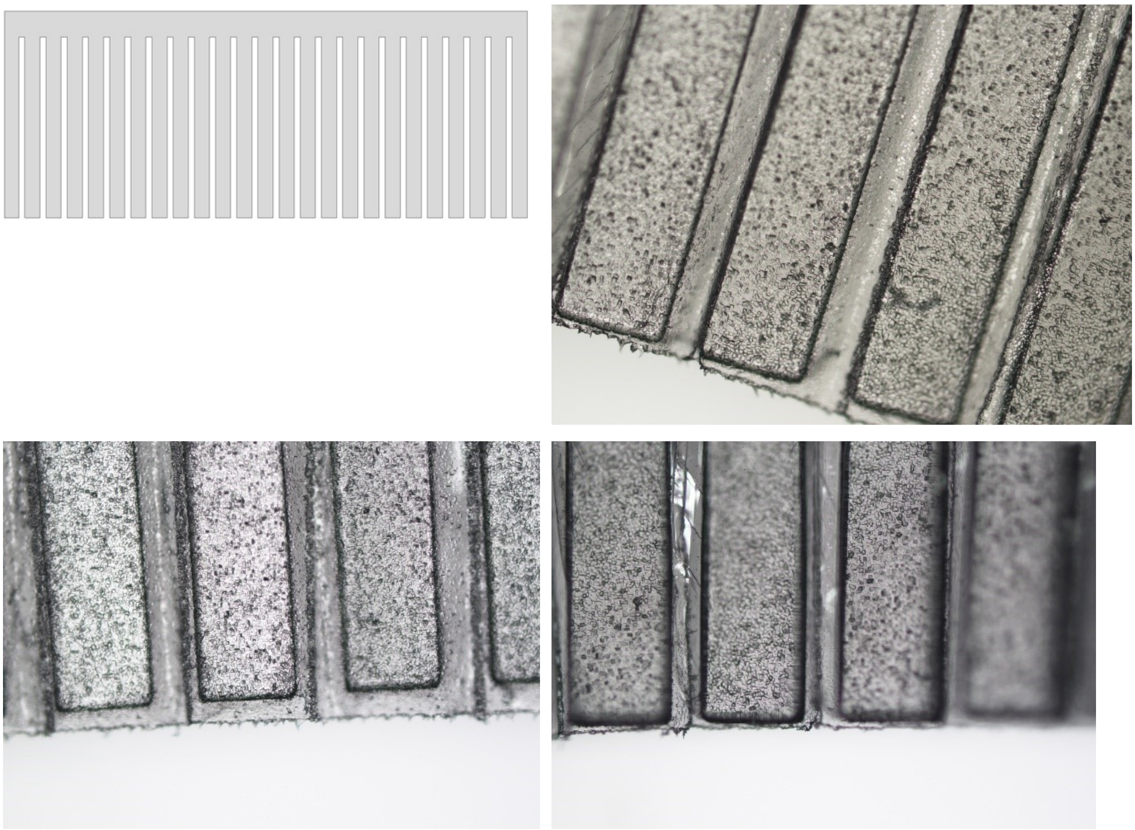

Figure S9: Degradation test: top left test specimen 17 mm x 6 mm x 0.2 mm, with 0.5 mm wide comb tooth; top right before ageing; bottom left after ageing with PBS and bottom right ageing with hydrogen peroxide.

Table S3 Data and results of the Accelerated Degradation Test

**PBS**		
**Wight before degradation**	**Wight after degradation**	**Percentual difference**
19.4	18.8	Discarded, because below minimum wight of the balance
19.4	19.2	
20.9	19.9	-4.30622
20.5	20.4	-0.4878
21.2	20.7	-2.35849
21.6	21.2	-1.85185
22.4	22.4	0

22.4	22.2	-0.89286
Mean ± standard error of mean		-1.6 ± 0.6
**3 % H_2O_2 solution**		
19.8	18.9	Discarded, because below minimum wight of the balance
19.5	19.3	
21.4	20.7	-3.27103
18.7	18.4	-7.35931
23.1	21.4	-0.9434
21.2	21	-2.21239
22.6	22.1	-4.01786
22.4	21.5	-3.27103
Mean ± standard error of mean		-3.6 ± 1.1

Mechanical properties

Flexural properties

Table S4: Measured flexural moduli for different mid layer thicknesses.

Designed mid layer thickness / µm	Determined flexural modulus / MPa
120	159 ± 9
165	136 ± 18
240	102 ± 9

The flexural properties of the sandwich material were measured using a custom-made apparatus consisting of a 500 mN load cell (Aurora Scientific 402B-HD) mounted ona motorised stage which moves the load cell vertically. A horizontal stainless-steel rod of radius 0.915 mm was fixed to the load cell to act as a testing probe. Rectangular samples (9 x 25 mm^2) of the material with three different mid layer thicknesses of 120 µm, 164 µm, and 240 µm were fabricated, and placed horizontally on two parallelly oriented support rods with a radius of 0.915 mm and a spacing L of 15 mm. The testing probe was pushed vertically onto the sampled with a constant speed of 10 mm·min^{-1}. Figure S10 shows a photograph of the setup.

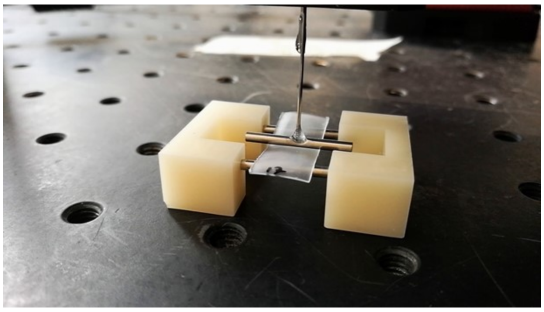

Figure S10: TOP: The rectangular samples are placed horizontally on two stainless steel rod supports. The load cell (not in the photo) with the rod shaped probe attachedto it is lowered from above by a motorised stage; BOTTOM: Nominal Stress-Strain Curve.

The real thicknesses *h* of the samples were measured prior to the experiment with a micrometer screw gauge. The strain *e* was calculated from the position *x* provided by the position encoder of the motorised stage by $e = 6xh/L^2$.[7] The stress σ was calculated according to $\sigma = 3FL/(2bh^2)$, where *F* is the force recorded by the load cell and *b* is the sample width.

The linear regimes of the resulting strain-stress curves were fitted and the flexural moduli which correspond to the slopes of the fitted linear functions were taken for each mid layer thickness. We determined for a mid-layer thickness of 120 µm a flexural modulus of 159 ± 9 MPa, for a thickness of 165 µm a modulus of 136 ± 18 MPa, and for a mid-layer thickness of 240 µm a flexural modulus of 102 ± 9 MPa. The measured flexural moduli are larger, i.e. the sandwich material is stiffer, than the flexural modulus of pure parylene C (4 MPa), but the measured flexural modulus is smaller, i.e. the sandwich material is more flexible, thanfor instance linear low density polyethylene 235 MPa to 800 MPa.[8]

Tensile properties

Table S5 summarises the determined Young's moduli and figure 1b shows the corres ponding data for selected samples.

Designed mid layer thickness / µm	Determined flexural modulus / MPa
120	71 ± 3
165	68 ± 5
240	38 ± 2

The tensile properties of the sandwich material were measured using a universal testing machine (Tinius Olsen 1ST) with a 25 N load cell (Tinius Olsen DBBMTOL-25N- 08-1002) and a laser extensometer (Tinius Olsen 500L). The same samples which were already characterised for their flexural properties were stretched longitudinally with a constant cross head speed of 10 mm/min and the applied force *F* and strain were recorded. The stress σ was calculated by $\sigma = F/(bh)$, where *b* is the sample width and *h* is the measured sample thickness[9]. The linear regions of the resulting stress strain curves were fitted with linear functions, where the Young'smoduli correspond to the slopes of the fits. We determined for a mid- layer thickness of 120 µm a Young's modulus of 71 ± 3 MPa, for a thickness of 165 µm a modulus of 68 ± 5 MPa, and for a mid- layer thickness of 240 µm a Young's modulus of 38 ± 2 MPa. These measured values are in between the Young's modulus of pure UV- cured Formlabs Elastic 50A resin 3.23 MPa and the Young's modulus of

[7] BS EN ISO 178:2019
[8] S. Singh, M.-C. Lo, V. B. Damodaran, H. M. Kaplan, J. Kohn, J. D. Zahn and D. I. Shreiber, *Sensors (Basel)*, 2016, **16**, 330; J. E. Mark, *Polymer Data Handbook*, Oxford University Press, 2009.
[9] BS EN ISO527-1:2019

Parylene- C (4.5 GPa).[10] With increasing thickness of the mid layer theproperties of the more stretchable 3D printer resin dominate over the less stretchable Parylene-C and hence the overall Young's modulus of the material decreases.

Pressure tests

In order to measure the maximum internal pressure, the microfluidic channels of the implant can sustain, blunt gauge 34 syringe needles were inserted into the inlets of four assembled implants and the connections were sealed with cyanoacrylate adhesive. The needles were then connected via an in-line pressure sensor (PendoTECH, PRESS-S- 000) to a syringe pump (KD scientific, Legato 110). Deionised water was pumped into the implant in a step-wise fashion (repetitions of 15 s continuous flow of 100 µL·min^{-1} followed by 15 s without flow) and the pressure was recorded. Figure S11 shows theacquired data. The maximum measured pressure in each device was interpreted as an approximation of the implant burst pressure. A mean burst pressure of 1.1 ± 0.1 bar was determined. Similar pressures were observed for aged samples. The same experiments were conducted with a the pro3dure GR-16, giving far lower burst pressures below 0.5 bar.

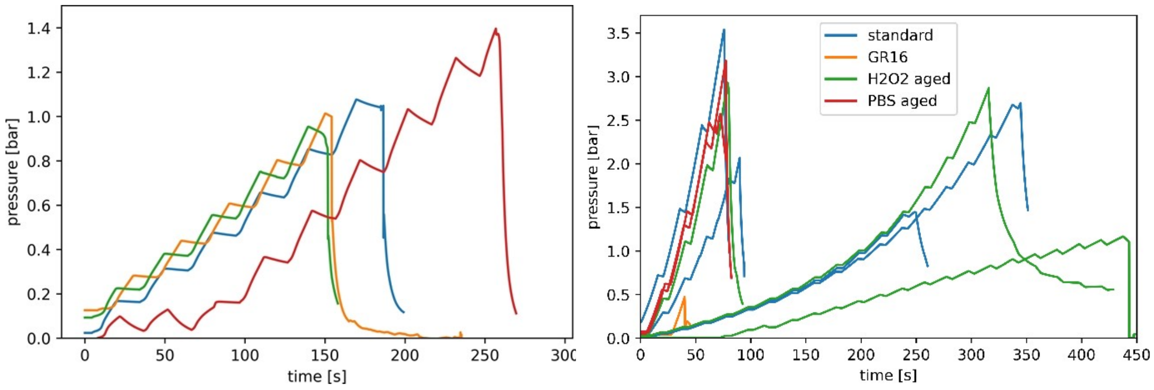

Figure S11 Burst Pressure Test: left Pressure versus time data of four implants. The non-zero pressures as zero time arise from pressure created when assembling the testing system before data acquisition started: right repeating of the standard measurement and comparison with aged samples as well as different resin (pro3dure GR 16).

Insertion force measurements

For brain implants, reduction of damage to surrounding brain tissue caused by device insertion is paramount. Therefore, the insertion force which is necessary to insert the implants into brain tissue has been measured. To investigate whether the tip angle of the implant has an impact on the insertion force gradient, the measurement was repeated for devices with tip angles of 10°, 20°, 30°, and 50°. As insertion body, 0.6 % m/v agarose (Merk, Agarose A9539) gels were chosen as it has been reported thatgels of this density resemble the physical properties of brain tissue.[11] The implants were fixed onto a 500 mN load cell

[10] https://formlabs-media.formlabs.com/datasheets/Elastic_Resin_Technical.pdf, 29.07.2021; 1. W. Sim, B. Kim, B. Choi and J. O. Park, *Microsystem Technologies*, 2005, **11**, 11-15.

(Aurora Scientific 402B-HD) which was mounted on a motorised stage. The implants were inserted into the gels with a constant speed of 10 mm·min^{-1} while the insertion force was recorded. Figure 6a shows the corresponding data and figure 6b visualises the setup. It should be noted that the implants were not completely flat but slightly bent before inserted into the gel which might have had an impact on the outcome of the measurements. The samples with a tip angle of 10° and 20° were less stiff than samples with a blunter tip angle which resulted in the implants bending away fromthe insertion direction by almost 90° inside the gel.

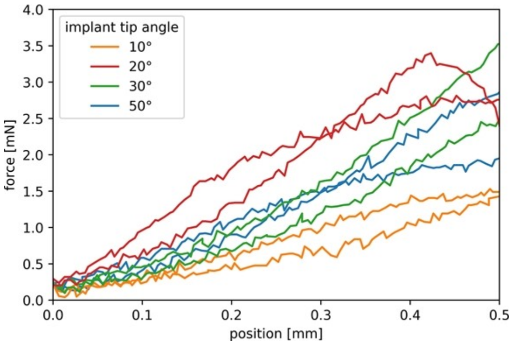

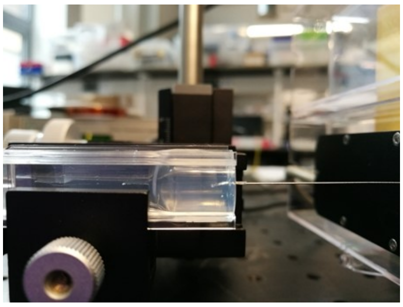

(a)

(b)

Figure S12: a) Insertion force versus implant position of implants with a tip angle of 10°,20°, 30°, and 50°. b) The implants (centre) were mounted onto a load cell (thin metal needle on the right) and inserted into plastic cuvettes filled with an agarose gel.

Electrochemical Impedance Spectroscopy EIS

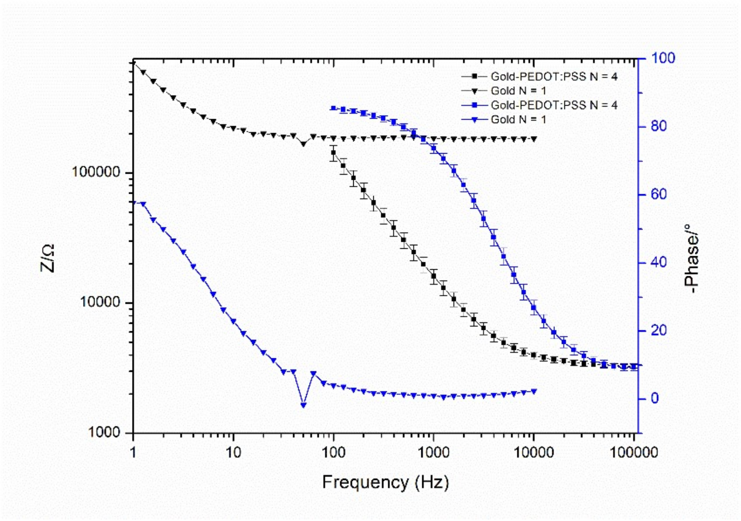

Figure S13 EIS recorded with 3 electrode configurations. Blue curves show the phase while the black curves show the impedance. The solid line represents the data of PEDOT coated gold electrodes, while the dashed lines represent pure gold electrodes. Error bars are the standard error of mean.

[11] R. Pomfret, G. Miranpuri and K. Sillay, *Ann Neurosci*, 2013, **20**, 118-122.

In vivo experiments

Animal surgery

All experimental procedures were performed in accordance with the UK Animals (Scientific Procedures) Act 1986. The work was approved by the University of Cambridge Animal Welfare and Ethical Review Body, and was conducted under the authority of a Project Licence (PFF2068BC). ~250 g Sprague Dawley rats (Charles River UK) were anaesthetised using isoflurane (2.5% in oxygen), and mounted on a stereotaxic frame. Their body temperature was monitored and maintained using a thermal blanket. A 1.5 mm^2 window was drilled into the skull, the dura carefully dissected, and the brain exposed. A hybrid fabricated probe was mounted onto the frame and gently lowered into the hippocampus CA1 (final coordinates for the tip of the probe: -4 mm anterio-posterior, 3 mm lateral, 3 mm depth).

For chronic implantations (tissue processing and immunohistochemistry), the space between the exposed brain and the body of the hybrid fabricated probe was covered in medical silicone (Kwik-cast silicone sealant, WPI). Two stainless steel screws (M0.8 x 2mm, US Micro Screw) were inserted into the skull, and the whole exposed skull and implant were sealed in place with surgical cement (Figure S14).

For electrophysiology recordings (electrophysiology recordings and drug delivery), a stainless steel screw was drilled into the CSF above the cerebellum to act as a recording ground. Isoflurane anaesthetic was lowered to 1.25 % during and prior to recordings.

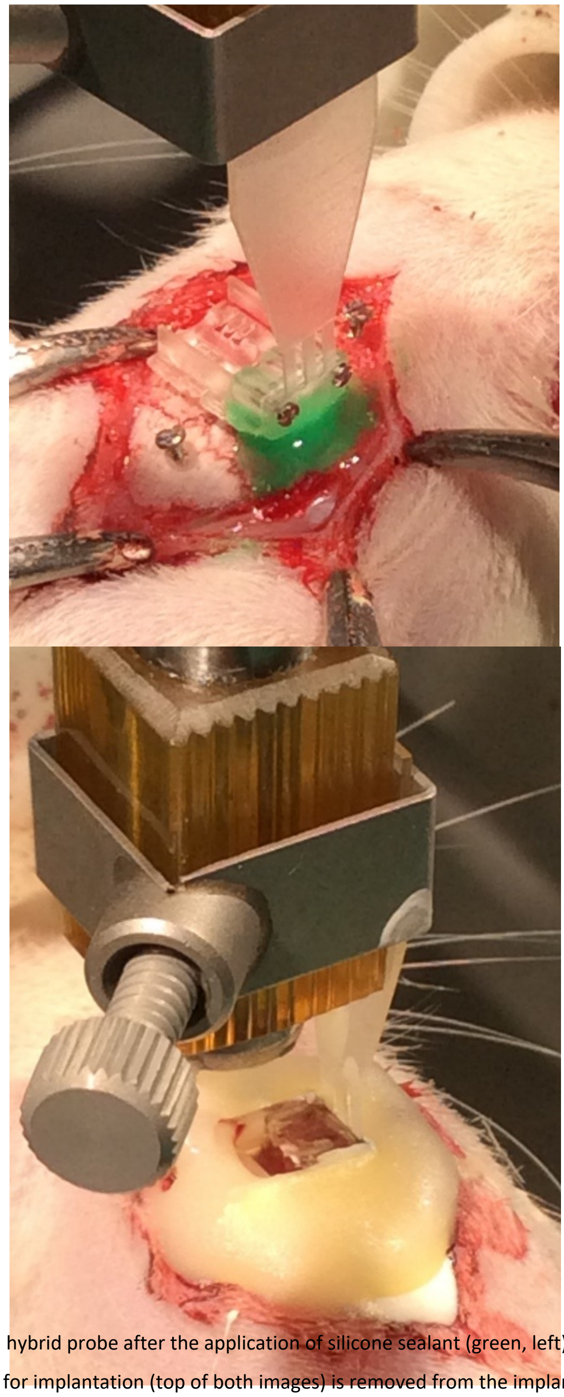

Figure S14. Image of implanted hybrid probe after the application of silicone sealant (green, left), followed by the application of surgical cement (right). Tab used for implantation (top of both images) is removed from the implant before the animal is allowed to recover.

Electrophysiology recordings and drug delivery

The hybrid fabricated probe was connected to electrophysiology recording hardware (RHS Stim/Recording headstage and controller, Intan Technologies), with the cerebellar screw acting as a ground. Voltage signals were recorded and amplified (X192), bandpass filtered between 1 Hz and 7.5 kHz, and digitised at

a 30 kHz sampling rate. To stimulate interictal-like activity, the RHS Stim/Record ground was connected to one of the electrodes in the hybrid fabricated probe. This formed a stimulating pair with one other electrode in the probe, locally stimulating the hippocampal tissue. The two most distant electrodes (bottom left and top right - Figure 1) were chosen to form this pair. Stimulation was carried out over 300 ms at 500 uA and 250 Hz (1 ms per pulse phase, followed 2 ms of interpulse period).

Local drug delivery was carried out by manually slowly injecting either 20 ul of 4AP (100 mM in phosphase buffered saline) or MPQX (54 uM in saline) over 30 s through one of the microfluidic channels of the hybrid fabricated probe.

For analysis, data was notch-filtered to remove mains noise and band-pass filtered between 1 and 400 Hz. Spikes were identified and counted when they had an amplitude larger than three standard deviations above the baseline using a custom-written script. Electrophysiology data analysis was carried out using Spike2 software (Cambridge Electronic Design, UK, v9.04b). Statistical analysis and data plotting was carried out in MATLAB (Mathworks, R2016b).

Tissue processing and Immunohistochemistry

Rats were humanely killed at 7 days post-implantation by exposure to a rising concentration of CO_2. Brains with probes still implanted were collected and transferred into 4% methanol-free paraformaldehyde in phosphate buffer saline with 0.1% sodium azide, and allowed to fix for 48 hours at 4ºC. Probes were then removed from fixed brains (Figure S15), and the brains were soaked in 30% of sucrose in phosphate buffered saline for cryoprotection for 72 hours. Brains were frozen and sectioned in a cryostat into 10 um sections, which were allowed to defrost and dry prior to immunostaining.

For staining, brain samples were blocked in 5% donkey serum in phosphate buffered saline with 0.1% sodium azide for 1 hour at room temperature. This was followed by incubation in primary Anti-GFAP antibody (ab7260) used at 1/1000 dilution in 5% foetal bovine serum in phosphate buffer saline with 0.1% sodium azide overnight at 4^oC. The next day samples were incubated in secondary antibodies (Donkey anti-Mouse IgG (H+L) Highly Cross-Adsorbed Secondary Antibody, Alexa Fluor 488 used at 1/2000 in 5% foetal bovine serum in phosphate buffer saline with 0.1% sodium azide) for 3 hours at room temperature in the dark. Tissue was then incubated in Vector TrueVIEW Autofluorescence Quenching Kit (Vector Laboratories) for 3 minutes at room temperature, followed by a wash in 5µg/mL Hoechst for 5 minutes. Between above incubation steps tissue was washed three times with 5% foetal bovine serum in phosphate buffered saline with 0.1%, 10 minutes per wash. Tissue was finally mounted and imaged in a Zeiss Axioscan Slide Scanner (Zeiss). Illumination intensity and exposure parameters were chosen such that GFAP+ astrocytes were visible in the naïve control brain samples.

For analysis, images of brain at 20X magnification were processed using a combination of custom-made Matlab (Mathworks, R2016b) and Fiji (v1.48, National Institutes of Health, USA) scripts. These produced a stain intensity profile at a range of depths from the edge of the implant, as indicated by the user in each image. This intensity was normalised against the stain intensity in a randomly-chosen 1 mm^2 area in the contralateral (non-implanted) brain hemisphere. Statistical analysis and data plotting was carried out in MATLAB (Mathworks, R2016b).

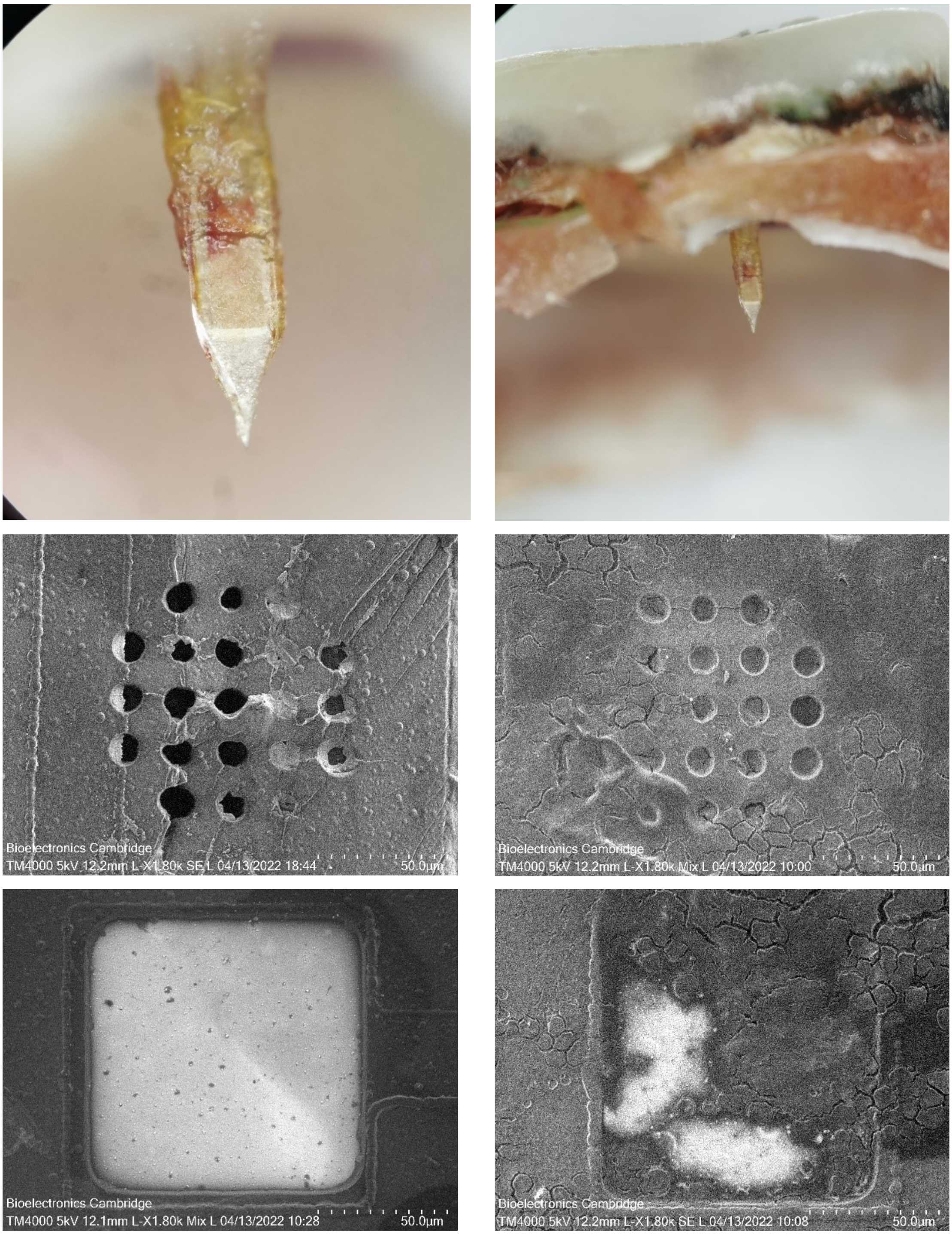

Figure S15. Pictures of test specimen and hybrid fabricated probe before and after 7 weeks implantation: top left: high magnification of test specimen after removal from fixed brain profile from side of skull; top left: test specimen still adhered to the skull *via* cement; middle left: SEM picture of the microfluidic outlet before and middle right: after 7 days implantation; bottom left: SEM picture of the electrode before and bottom right: after 7 days implantation.

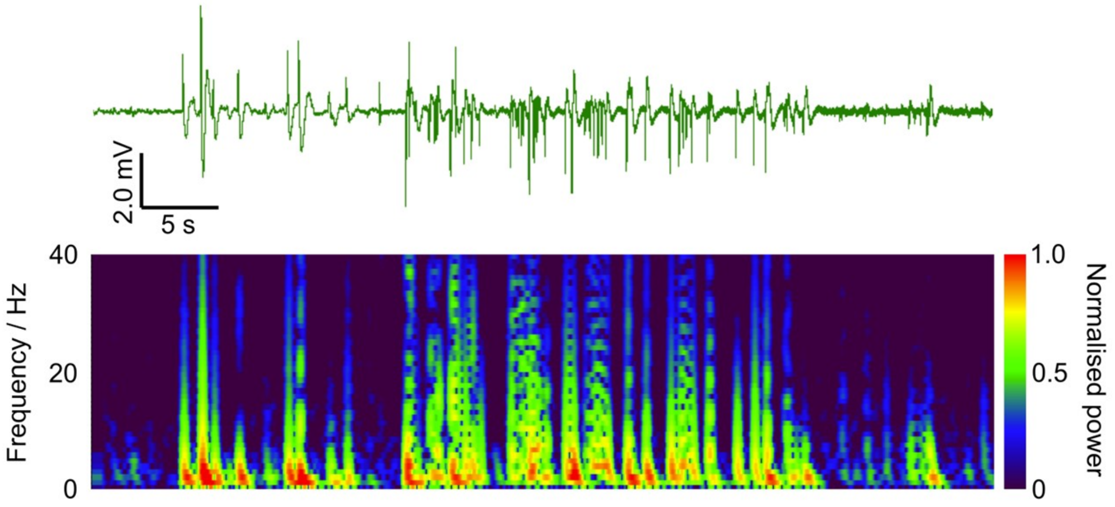

Figure S16. Hippocampal recorded trace (top) and corresponding time/frequency plot (bottom) following delivery of 4AP. Generated using a 32768 FFT block size and a Hanning window.
